# Supplementary material for: Estimating the impact of city-wide Aedes aegypti population control: An observational study in Iquitos, Peru
Source: PLoS Negl Trop Dis. 2019 May 30;13(5):e0007255. doi: 10.1371/journal.pntd.0007255 (PMC6542505; doi:10.1371/journal.pntd.0007255)
Supplement: S1 Text — (DOCX) [file pntd.0007255.s001.docx]

### Supporting Information

Results from the base model confirmed that there was statistically significant over-dispersion (Poisson model AIC: 291417, negative binomial model AIC: 194781). The base model (with only the offset as a covariate) performed poorly in some cases, with the Poisson model explaining 0% of the deviance and the negative binomial model explaining 3.9%. Including an additional two-dimensional smooth representing abiotic/unexplained spatial variation significantly improved fit (Poisson model AIC: 287633, negative binomial model AIC: 196340). The addition of the two abiotic temporal smooths further increased fit significantly (Poisson model AIC: 280361, negative binomial model AIC: 193940). For comparison purposes, a model without the spatial smooth, but with the two temporal smooths was fit as well. The model with only the temporal variation accounted for performed better that the model with the spatial smooth (Poisson model AIC: 284082 vs 287633, negative binomial model AIC: 195178 vs 196340).

Among the temperature covariates, the model that included minimum temperature performed better than when mean or maximum temperature were included (negative binomial AIC of 192539 versus 192588 and 192857, respectively). The full model increased the deviance explained considerably (5.6% and 10.3% respectively). The ‘drop one’ test did not identify any covariates that should be dropped (Table 1). Judged by the change in AIC, the least influential covariate for the negative binomial model was daily temperature range, with a drop in fit corresponding to an increase in AIC of 95. The most influential covariate was maximum wind speed, with a drop in fit corresponding to an increase in AIC of 1721.

The inclusion of the control covariate further improved the fit. Inspection of the fitted SCAM model revealed that the smooth for control effort was found to be linear. To simplify the model, we reverted to the GAM model with control effort entering linearly. For the negative binomial model, the final model with control effort linearly added to the reduced model, improving AIC by a further 144 points.

To show the relationship between any given covariate and the response for all plots from the final model, we held all other covariates constant. Unless otherwise indicated, all plots represent the abundance in the center of the city on 28 April 2001, with all meteorological drivers set to their mean and no control assumed. This date was chosen because it was estimated to have the highest expected abundance.

The final model clearly captured variation in space (Figure 5, Figure S11). On 28 April 2001, the range of expected mosquitoes found per visit varied from almost 0.5 up to 3 with the highest expected abundance in the far north. There was a greater coefficient of variation on the periphery of the city, with a noticeable increase in the north where the greatest expected mosquito counts occurred. Within-year variation (Fig 6b) showed clear seasonality, with the most mosquitoes expected late in the year and the fewest in June and July. These dates matched the timing of the dry and wet seasons in Iquitos. After controlling for the influence of weather, 2002 was the year with the lowest mosquito abundance (Figure 6a). Wind speed had a strongly non-linear relationship with abundance, with higher counts typically observed on windier days (Figure S1).

The DLNM relationships were each plotted in a similar manner to each other. In the left panel, the z-axis shows the multiplicative effect of various combinations of lag and weather value. The lag axis shows the effect of weather up to 30 days in the past, while the front axis shows the range of values of the covariate. For each of these figures, interpretation requires envisioning what the value of the covariate was over the course of the past 30 days and then averaging over this curve on the surface, somewhat analogous to line integrals. The predicted values were all centered on the mean meteorological value, so each surface is constant at 1 for the mean meteorological value across lags. For the remaining 6 panels, slices of the surface were taken and plotted with 95% confidence intervals. The center panels display the relationship across different lags between the meteorological variable at the lowest, middle, and highest values observed and expected mosquito counts. These represent the expected relationship between the meteorological driver and mosquito abundance when the meteorological driver was held constant at that value over the past 30 days. The right panels slice the DLNM surface across lags of 0, 10, and 20 days. These represent the relationship between the meteorological driver and mosquito abundance at the moment of capture as well as 10 and 20 days in the past. Again, for the right panel, the relationship was plotted using the mean meteorological value as the reference value. These relationships go through 1 with a vanishing confidence interval at that point.

For the full model, it is important to note that identified relationships are different than in univariate meteorological models due to collinearity in the meteorological covariates. The variation captured by each meteorological driver must be interpreted in the presence of all other drivers. For minimum temperature (Figure S2), there was an almost quadratic relationship between lag and response for low temperatures and essentially no multiplicative effect for high temperatures across lags. Precipitation was skewed right with the mean value of 0.96. As such, the reference value was close to the lowest observed value and thus the top center panel of Figure S3 shows no multiplicative effect by construction. Conversely, large rainfall a month in the past resulted in an increased expected count. Similarly, large rainfalls 2 weeks before capture reduced expected abundance. For daily temperature range (Figure S4), large variation in daily temperature 30 days in the past increased the expected number of mosquitoes caught. For low variations in daily temperature, there was only a small effect. Recall that in the full model, daily temperature range was the covariate closest to being dropped. The relationship between relative humidity (Figure S5), time, and expected mosquitoes caught was complex. Iquitos is in the Amazon Basin where the environment is quite humid throughout the year; relative humidity ranges from 61% to almost 100%. ‘Moderate’ humidity of 84.6% was used as the baseline because this was the mean estimated daily relative humidity and thus had no multiplicative effect on mosquito abundance. Either ‘drier’ or wetter environments may increase or decrease abundance depending on when in the development stage of the mosquito the weather changes.

For the univariate meteorological models, relationships changed between each driver and response. These models were fit with all non- meteorological drivers and then only a single meteorological driver at a time. This allowed for a more direct interpretation of the weather variable’s influence on mosquito abundance. When minimum temperature and abundance were assessed in isolation (Figure S6), the relationship more closely matched the expectation derived from survival studies [43]. At both high and low temperatures, abundance was expected to decrease relative to moderate temperatures. That relationship was mostly constant in lag space, with only substantial curvature at extremely low temperatures, where expected counts were already decreased in all cases. The relationship between precipitation and abundance also changed from the full model. At a lag of 0, there was a mostly monotonically increasing relationship between precipitation and expected abundance, while at earlier lags this relationship was reduced (Figure S7). Daily temperature’s relationship to abundance switched in some cases (Figure S8) relative to the multivariate analysis with high variation in daily temperature a month in the past, resulting in a large decrease in expected mosquito abundance. For relative humidity (Figure S9), at moderate lags the relationship followed expectation, with higher humidity resulting in higher abundance. At no lag, the relationship was quadratic, with a decrease in expected abundance at both high and low relative humidity levels. In this context, ‘low’ is still 61% relative humidity.

Figures

**Figure S1: Effect of max wind speed on mosquito abundance**. Expected number of female *Ae. aegypti* captured with 95% uncertainty as a function of wind speed on a home in the center of the city measured on April 28^th^, 2001. These choices (home in the center of the city, the date of April 28^th^, 2001) are all arbitrary but necessary to produce estimated counts that account for spatial and temporal variation.

**Figure S2: Effect of lagged daily minimum temperature on mosquito abundance in the full model.** Panel A: The relationship between minimum temperature (x-axis), lag up to 30 days (y-axis), and relative rate of number of mosquitoes caught (z-axis) is plotted. Panel B, D, and F plot slices of the surface along the temperature axis with corresponding uncertainty. Panel C, E, and G plot slices of the surface along the lag axis with corresponding uncertainty.

**Figure S3: Effect of lagged daily precipitation on mosquito abundance in the full model.** Panel A: The relationship between precipitation (x-axis), lag up to 30 days (y-axis), and relative rate of number of mosquitoes caught (z-axis) is plotted. Panel B, D, and F plot slices of the surface along the temperature axis with corresponding uncertainty. Panel C, E, and G plot slices of the surface along the lag axis with corresponding uncertainty.

**Figure S4: Effect of lagged daily temperature range on mosquito abundance in the full model.** Panel A: The relationship between daily temperature range (x-axis), lag up to 30 days (y-axis), and relative rate of number of mosquitoes caught (z-axis) is plotted. Panel B, D, and F plot slices of the surface along the temperature axis with corresponding uncertainty. Panel C, E, and G plot slices of the surface along the lag axis with corresponding uncertainty.

**Figure S5: Effect of lagged daily relative humidity on mosquito abundance in the full model.** Panel A: The relationship between relative humidity (x-axis), lag up to 30 days (y-axis), and relative rate of number of mosquitoes caught (z-axis) is plotted. Panel B, D, and F plot slices of the surface along the temperature axis with corresponding uncertainty. Panel C, E, and G plot slices of the surface along the lag axis with corresponding uncertainty.

**Figure S6: Effect of lagged daily minimum temperature on mosquito abundance in a univariate model.** Panel A: The relationship between minimum temperature (x-axis), lag up to 30 days (y-axis), and relative rate of number of mosquitoes caught (z-axis) is plotted. Panel B, D, and F plot slices of the surface along the temperature axis with corresponding uncertainty. Panel C, E, and G plot slices of the surface along the lag axis with corresponding uncertainty.

**Figure S7: Effect of lagged daily precipitation on mosquito abundance in a univariate model.** Panel A: The relationship between precipitation (x-axis), lag up to 30 days (y-axis), and relative rate of number of mosquitoes caught (z-axis) is plotted. Panel B, D, and F plot slices of the surface along the temperature axis with corresponding uncertainty. Panel C, E, and G plot slices of the surface along the lag axis with corresponding uncertainty.

**Figure S8: Effect of lagged daily temperature range on mosquito abundance in a univariate model.** Panel A: The relationship between daily temperature range (x-axis), lag up to 30 days (y-axis), and relative rate of number of mosquitoes caught (z-axis) is plotted. Panel B, D, and F plot slices of the surface along the temperature axis with corresponding uncertainty. Panel C, E, and G plot slices of the surface along the lag axis with corresponding uncertainty.

**Figure S9: Effect of lagged daily relative humidity on mosquito abundance in a univariate model.** Panel A: The relationship between relative humidity (x-axis), lag up to 30 days (y-axis), and relative rate of number of mosquitoes caught (z-axis) is plotted. Panel B, D, and F plot slices of the surface along the temperature axis with corresponding uncertainty. Panel C, E, and G plot slices of the surface along the lag axis with corresponding uncertainty.

**Figure S10: Schematic of the functional form for the impact of intervention coverage on the relative abundance of mosquitoes.** The multiplicative effect of the intervention effort reduces mosquitoes from baseline from the beginning of the intervention round through 3 weeks past the end of the intervention round. If two intervention rounds overlap, the effect is assumed to be the larger of each individual impacts and not additive. The SCAM component of the model fits the function $f(x)$.

**Figure S11: Fitted spatial variation in female *Ae. aegypti* abundance.** Panel A: Estimated number of female *Ae. aegypti* that would be caught during a house-hold aspiration held on April 28^th^, 2001 displayed as a contour plot. The choice of this date was arbitrary but necessary to incorporate the meteorological covariates. Panel B: Estimated coefficient of variation on number of mosquitoes for April 28^th^, 2001 displayed as a contour plot.

**Figure S12: Profile likelihood for the offset.** Using the base model, the offset was varied from its maximum likelihood estimate to identify the profile likelihood confidence interval.

**Figure S13: Relationship between holdout fraction of data and estimated effect of space spraying.** The estimated effect of space spraying is plotted across 100 experiments for each level of holdout (retaining between 5% and 80% of the original data). The left y-axis shows the fitted value in log space, while the right y-axis displays it in natural space. The dashed line indicates the fitted value of the final model.

**Figure S14: Relationship between holdout fraction of data and estimated standard error of effect of space spraying.** The estimated standard error of the effect of space spraying is plotted across 100 experiments for each level of holdout (retaining between 5% and 80% of the original data). The dashed line indicates the fitted standard error of the final model.

**Figure S15: Log fit versus log observed data.** Observations and fitted values are logged (after adding one to each observation) and plotted against each other.

**Movie S1: Predicted abundance of mosquitoes through space and time.** Figure 5a is replotted for the first day of each week across the study period. The time-series is representative of the single point in the South-West of Iquitos indicated by an open circle on the 3-D surface.
