# Supplementary material for: Estimating the impact of city-wide Aedes aegypti population control: An observational study in Iquitos, Peru
Source: PLoS Negl Trop Dis. 2019 May 30;13(5):e0007255. doi: 10.1371/journal.pntd.0007255 (PMC6542505; doi:10.1371/journal.pntd.0007255)
Supplement: S1 Table — Project titles and descriptions are listed for each study conducted in Iquitos, Peru that contributed to the entomological surveys. Associated duration, protocol numbers, and IRBs are also listed, as well as primary publications that describe the primary results of those studies. (DOCX) [file pntd.0007255.s002.docx]

Table 1: Summary of Entomological Surveys conducted between 1998 and February 2011 used to construct mosquito surface.

| Project Title and Description | Duration | Protocol No.’s* | IRBs** | Publications |
| --- | --- | --- | --- | --- |
| **Entomological correlates to dengue transmission**  Geographically stratified sample of 15 city blocks in each of 8 regions of Iquitos City, to include all households on blocks containing human cohort members, forming a sampling circuit of ≈ 6,000 households (range= 5,721-6,466) from the same areas of the city, divided into 17 groups. Within a circuit, each house was sampled once. With the exception of the first sampling circuit (9 months) the entire circuit was sampled at 4 month intervals (13 consecutive times). In 1998, 2 consecutive surveys of 600 contiguous houses in two Iquitos neighborhoods (Maynas, Tupac Amaru) were carried out. | 1999-2003 | 234832  NMRCD.2001.0008  ID-013-2002/INS | UCDAVIS  NMRC  INS | [1-5] |
| **A Two-Year Study To Evaluate The Dengue Vector Control System (DVCS) in Peru**  26 city blocks, geographically distributed, pre-intervention period April 2004-January 2005, with no vector control, February 2005-February 2006, 12 block received 1-time source reduction followed by 1time residual spray with CS-Demand (Lambdacyhalotrin) and monthly applications of BTI to water containers. All blocks received 3 cycles of space sprays in December 2004. All households on study blocks were surveyed at 2 month intervals. | 2004-2006 | 743851  NMRCD.2003.0008  ID-045-2003/INS  WRAIR | UCDAVIS  NMRC  INS | [6] |
| **Multi-country study on the pupal survey technique for the Dengue Vector *Aedes aegypti***  Non-residential sites were surveyed twice over a one-year period, including houses around a large open air market and commercial areas in Iquitos. Sites included markets, ports, factories, machine shops, lumberyards, recreation areas, and government buildings. | 2004-2005 |  | Waiver | [7, 8] |
| **Outbreak Investigation of Venezuelan Equine Encephalitis Virus (VEEV)**  Entomological surveys were carried out in 3 areas of these city based on the residences of VEEV-infected patients. The neighborhoods included in this study were Bella Vista Nanay located in the northern-most section of the city; Belén, located in the eastern part of the city along the Itaya River; and three sites in the Southern San Juan District. This was a one time survey carried out in October 2006. | 2006 |  | Waiver | [9] |
| **Efficacy and implementation of two Targeted control strategies to control the Dengue Vector *Aedes aegypti***  Five city blocks from 10 Ministry of Health (MOH) Zones selected at random. Study divided into Pre- (Jan 2006-Februrary 2007) and Post-intervention (after February 2007) periods, One block from each MOH zone was assigned to 1 of the 5 following interventions: 92% targeted control with pyriproxyfen, 58% targeted control with pyriproxyfen, non-targeted larval control with pyriproxyfen, temephos control (2 month intervals) and temephos control (3 month intervals). Houses were surveyed then treated according to treatment starting in March-April 2007 at approximately 2 month intervals. *Ae. aegypti* surveys carried out at through January 2008. A large city-wide ULV door to door space spray campaign was carried out in March 2008. In addition a acceptability/cost efficiency trial was conducted in 652 contiguous households in the San Juan District of Iquitos. From July 27-August 6 pre-intervention surveys were carried out, followed by post-interevention (92% targeted control versus standard MOH larviciding) from August 7-15, 2007. | 2006-2008 | 281363  NMRCD2007.001 | UCDAVIS  NMRCD | [10] |
| **Measuring Entomological Risk for dengue**  Entomological surveys carried out in two Iquitos neighborhoods (Maynas [~20 city blocks] and Tupac Amaru [14 city blocks]) of ~900 houses each. All open households were surveyed at approximately 4 month intervals. In addition to neighborhood houses, multiple individual houses reported as visited by neighborhood cohort members were surveyed at the same 4 month intervals. Study area only received standard MOH vector control. | 2008-2013 | 2007.15244  NMRCD2007.007  9162  Relying Agreement | UCDAVIS  NAMRU-6  EMORY  TULANE  SDSU | [11] |
| **Can Insecticide Treated Curtains Prevent Dengue Transmission**  Randomized Cluster Trial designed to test the efficacy of insecticide-treated curtains placed in 10 of 20 clusters containing a minimum of 70 households each. Clusters were contiguous and the study area was located in the district of San Juan in the southern most region of urban Iquitos. Entomological surveys were attempted in all households within each cluster prior to curtain deployment (October 2009), within 1-2 months after deployment (January 2010), with subsequent surveys in May 2010, February 2011 and May-June 2011. Curtain failure was observed in the May 2010 and the curtains retreated with deltametrithn in November 2010. | 2009-2011 | NMRCD2009.007  Relying Agreement | NAMRU-6  UCDAVIS  LSTM  LSTMH | [12, 13] |

*IRB changed numbering systems of protocols overtime, we provide the most up to date number of the protocol.;**UCDAVIS=University of California, Davis, NMRC=Naval Medical Research Center, INS=Instituto Nacional de Salud (Peruvian National Institute of Health, WRAIR=Walter Reed Army Institute of Research, NMRCD=Naval Medical Research Center Detachment, Lima, Peru; EMORY=Emory University, TULANE=Tulane University; LSTM=Liverpool School of Tropical Medicine; LSTMH=London School of Tropical Medicine and Hygeien..

1. Morrison AC, Gray K, Getis A, Astete H, Sihuincha M, Focks D, et al. Temporal and geographic patterns of Aedes aegypti (Diptera: Culicidae) production in Iquitos, Peru. J Med Entomol. 2004;41(6):1123-42. Epub 2004/12/21. PubMed PMID: 15605653.

2. Morrison AC, Minnick SL, Rocha C, Forshey BM, Stoddard ST, Getis A, et al. Epidemiology of dengue virus in Iquitos, Peru 1999 to 2005: interepidemic and epidemic patterns of transmission. PLoS Negl Trop Dis. 2010;4(5):e670. Epub 2010/05/11. doi: 10.1371/journal.pntd.0000670. PubMed PMID: 20454609; PubMed Central PMCID: PMC2864256.

3. Getis A, Morrison AC, Gray K, Scott TW. Characteristics of the spatial pattern of the dengue vector, Aedes aegypti, in Iquitos, Peru. Am J Trop Med Hyg. 2003;69(5):494-505. Epub 2003/12/26. PubMed PMID: 14695086.

4. Morrison AC, Astete H, Chapilliquen F, Ramirez-Prada C, Diaz G, Getis A, et al. Evaluation of a sampling methodology for rapid assessment of Aedes aegypti infestation levels in Iquitos, Peru. J Med Entomol. 2004;41(3):502-10. Epub 2004/06/10. PubMed PMID: 15185957.

5. Morrison AC, Astete H, C. R, H. R, G.A. S, F. A, et al. Evaluation of Emergency Vector Control Measures during a Dengue Epidemic in Iquitos, Peru 2002-2003. American Journal of Tropical Medicine and Hygiene. 2003;Abstract.

6. Morrison A, Astete H, Rocha C, Lopez V, Olson J, Kochel T, et al. Impact of the dengue vector control system (DVCS) on Aedes aegypti populations in Iquitos, Peru 2004-2005. American Journal of Tropical Medicine and Hygiene. 2005;73(6):326-. PubMed PMID: WOS:000202990001415.

7. Morrison AC, Sihuincha M, Stancil JD, Zamora E, Astete H, Olson JG, et al. Aedes aegypti (Diptera: Culicidae) production from non-residential sites in the Amazonian city of Iquitos, Peru. Ann Trop Med Parasitol. 2006;100 Suppl 1:S73-S86. Epub 2006/04/25. doi: 10.1179/136485906X105534. PubMed PMID: 16630393.

8. Alexander N, Lenhart AE, Romero-Vivas CM, Barbazan P, Morrison AC, Barrera R, et al. Sample sizes for identifying the key types of container occupied by dengue-vector pupae: the use of entropy in analyses of compositional data. Ann Trop Med Parasitol. 2006;100 Suppl 1:S5-S16. Epub 2006/04/25. doi: 10.1179/136485906X105471. PubMed PMID: 16630387.

9. Morrison AC, Forshey BM, Notyce D, Astete H, Lopez V, Rocha C, et al. Venezuelan equine encephalitis virus in Iquitos, Peru: urban transmission of a sylvatic strain. PLoS Negl Trop Dis. 2008;2(12):e349. Epub 2008/12/17. doi: 10.1371/journal.pntd.0000349. PubMed PMID: 19079600; PubMed Central PMCID: PMC2593782.

10. Tun-Lin W, Lenhart A, Nam VS, Rebollar-Tellez E, Morrison AC, Barbazan P, et al. Reducing costs and operational constraints of dengue vector control by targeting productive breeding places: a multi-country non-inferiority cluster randomized trial. Trop Med Int Health. 2009;14(9):1143-53. Epub 2009/07/25. doi: TMI2341 [pii]

10.1111/j.1365-3156.2009.02341.x. PubMed PMID: 19624476.

11. LaCon G, Morrison AC, Astete H, Stoddard ST, Paz-Soldan VA, Elder JP, et al. Shifting patterns of Aedes aegypti fine scale spatial clustering in Iquitos, Peru. PLoS Negl Trop Dis. 2014;8(8):e3038. doi: 10.1371/journal.pntd.0003038. PubMed PMID: 25102062; PubMed Central PMCID: PMCPMC4125221.

12. Paz-Soldan VA, Bauer K, Morrison AC, Cordova Lopez JJ, Izumi K, Scott TW, et al. Factors Associated with Correct and Consistent Insecticide Treated Curtain Use in Iquitos, Peru. PLoS Negl Trop Dis. 2016;10(3):e0004409. doi: 10.1371/journal.pntd.0004409. PubMed PMID: 26967157; PubMed Central PMCID: PMCPMC4788147.

13. Paz-Soldan VA, Bauer KM, Lenhart A, Cordova Lopez JJ, Elder JP, Scott TW, et al. Experiences with insecticide-treated curtains: a qualitative study in Iquitos, Peru. BMC Public Health. 2016;16:582. doi: 10.1186/s12889-016-3191-x. PubMed PMID: 27422403; PubMed Central PMCID: PMCPMC4947330.
