# Supplementary figures and images for: Estimating the impact of city-wide Aedes aegypti population control: An observational study in Iquitos, Peru
Source: PLoS Negl Trop Dis. 2019 May 30;13(5):e0007255. doi: 10.1371/journal.pntd.0007255 (PMC6542505; doi:10.1371/journal.pntd.0007255)

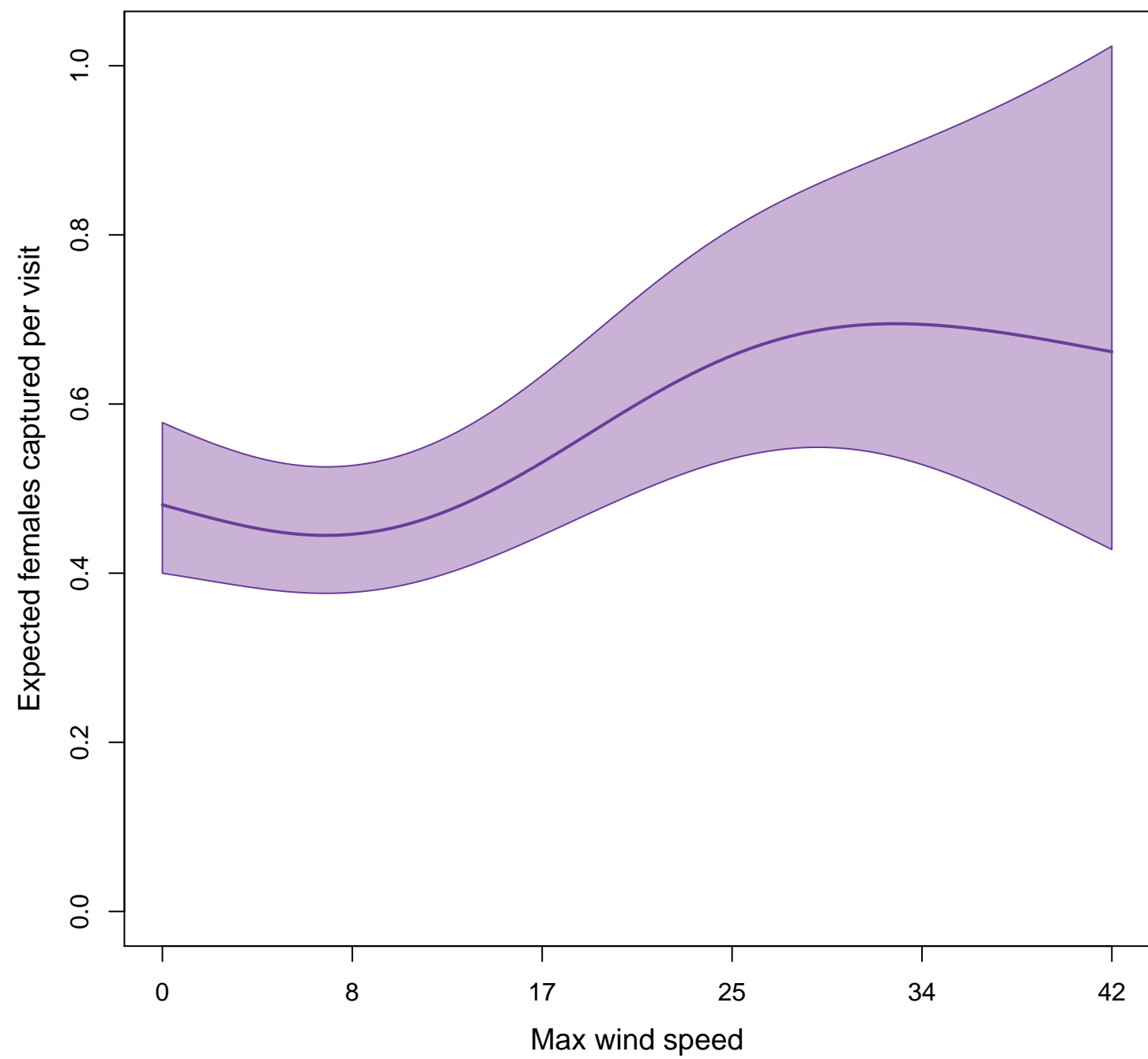

Supplement: S1 Fig — Expected number of female Ae. aegypti captured with 95% uncertainty as a function of wind speed on a home in the center of the city measured on April 28th, 2001. These choices (home in the center of the city, the date of April 28th, 2001) are all arbitrary but necessary to produce estimated counts that account for spatial and temporal variation. (PDF) [file pntd.0007255.s003.pdf]

3D graph of Precipitation effect

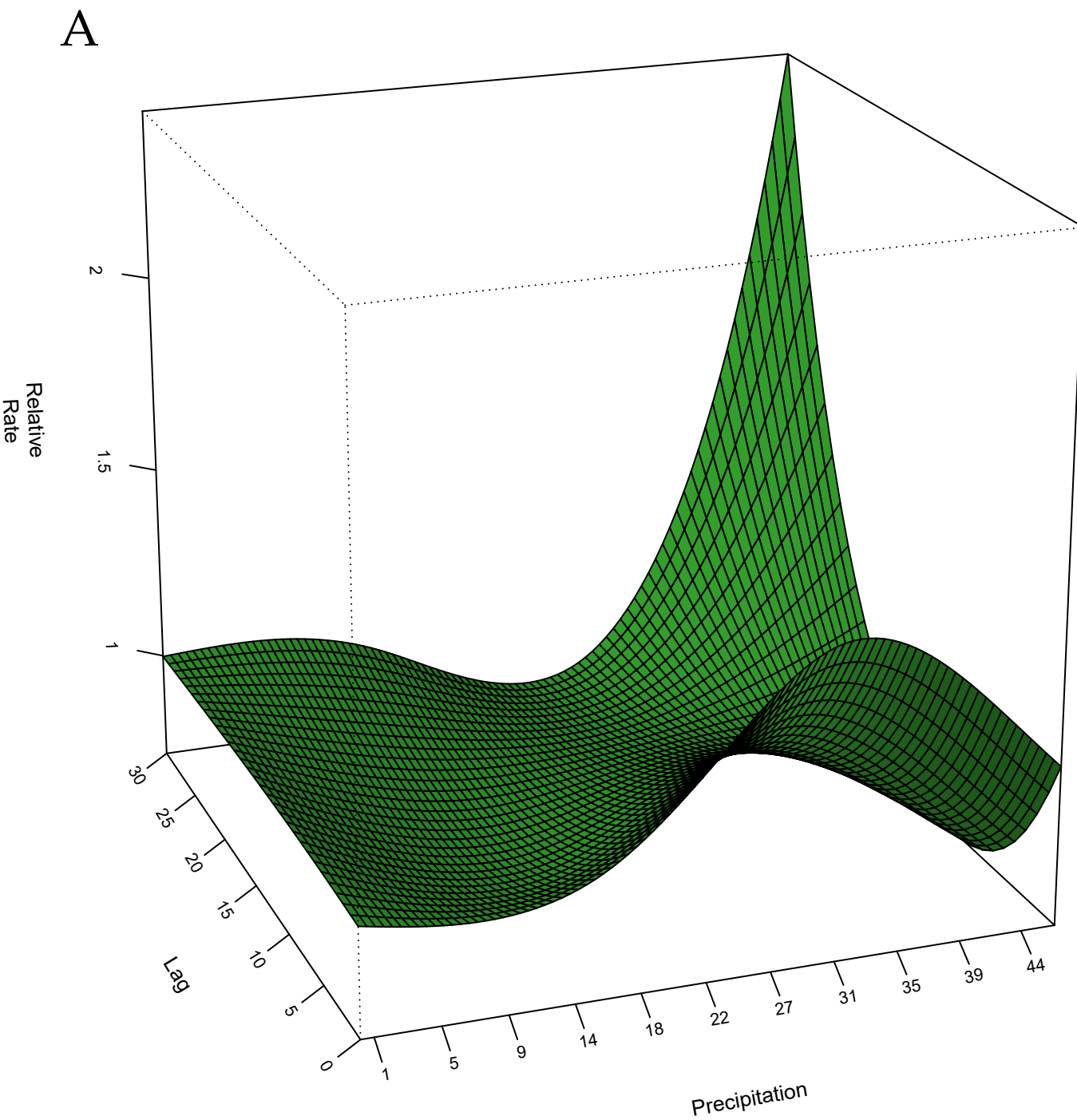

Slices in Precipitation dimension

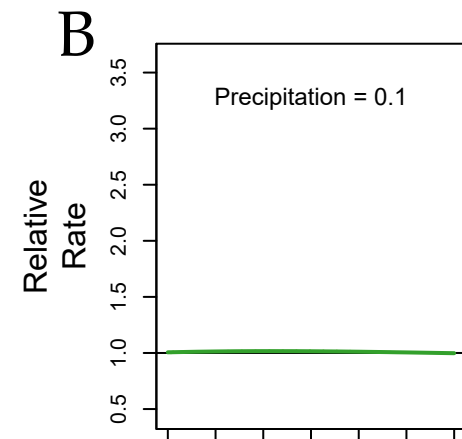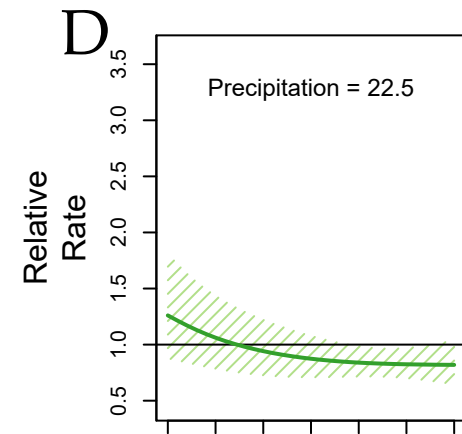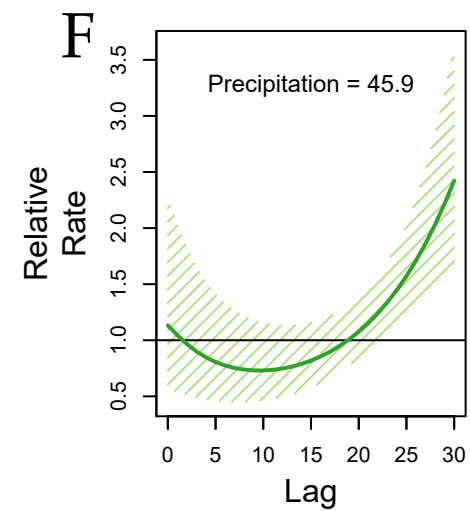

Slices in lag dimension

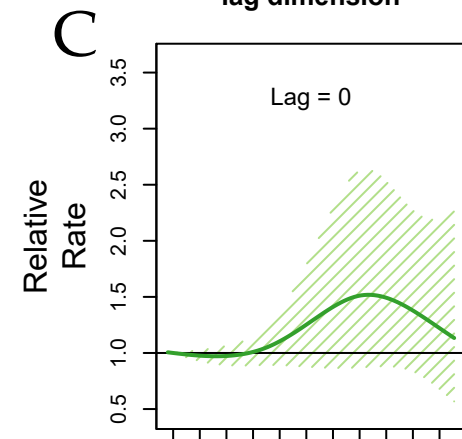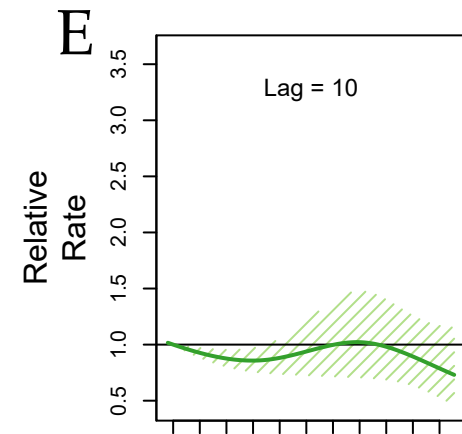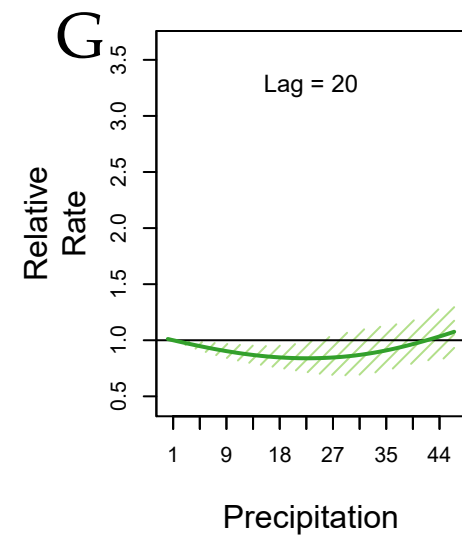

Supplement: S3 Fig — Panel A: The relationship between precipitation (x-axis), lag up to 30 days (y-axis), and relative rate of number of mosquitoes caught (z-axis) is plotted. Panel B, D, and F plot slices of the surface along the temperature axis with corresponding uncertainty. Panel C, E, and G plot slices of the surface along the lag axis with corresponding uncertainty. (PDF) [file pntd.0007255.s005.pdf]

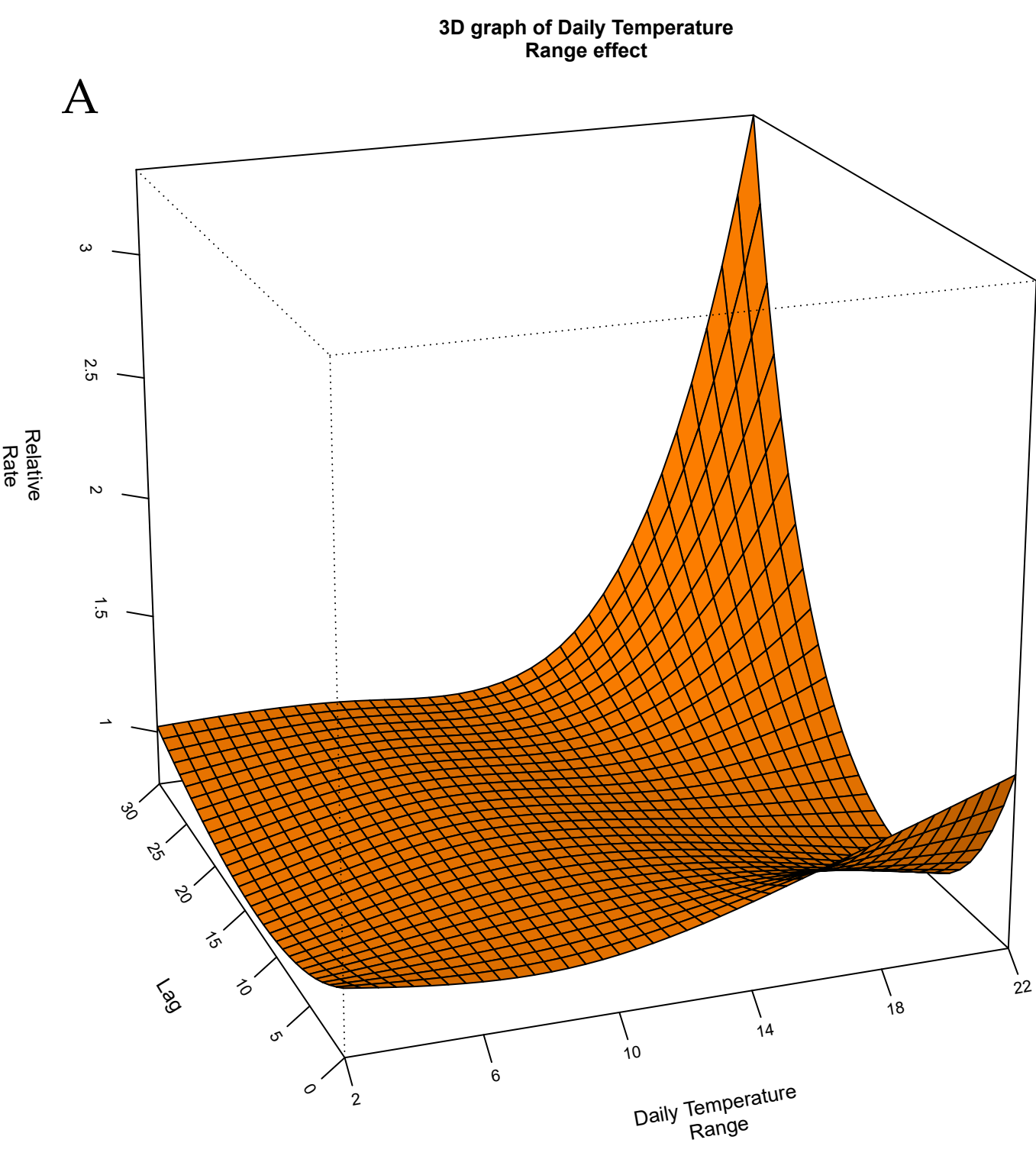

**B**

Slices in Daily Temperature Range dimension

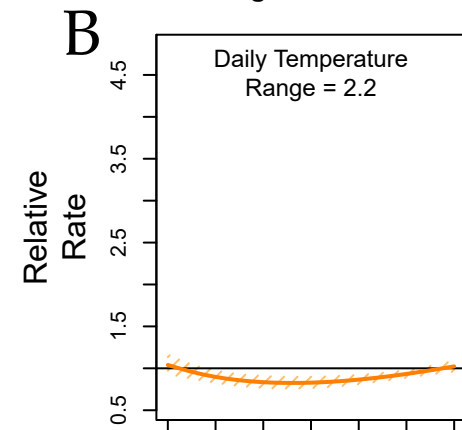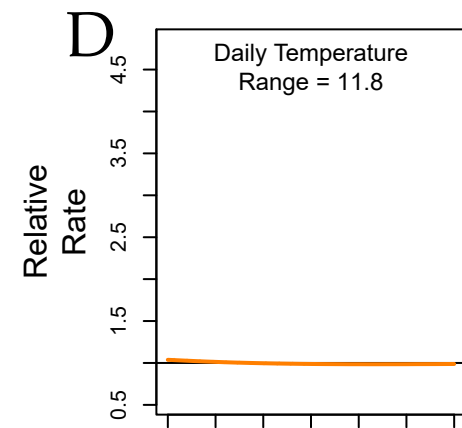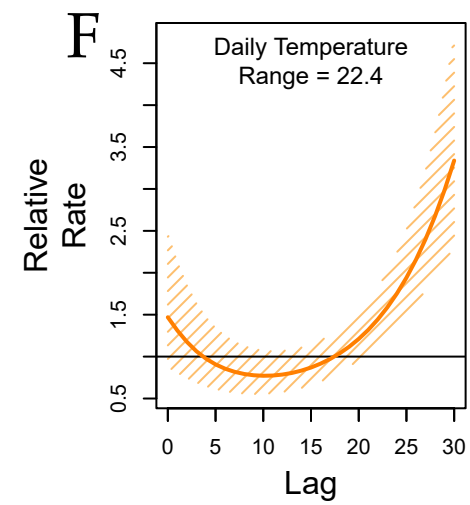

**C**

Slices in lag dimension

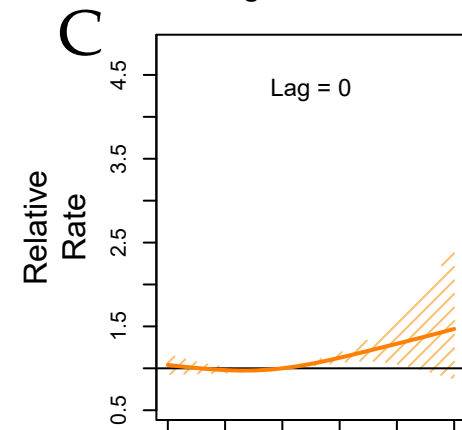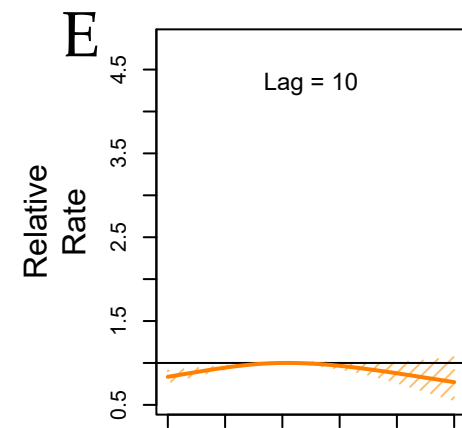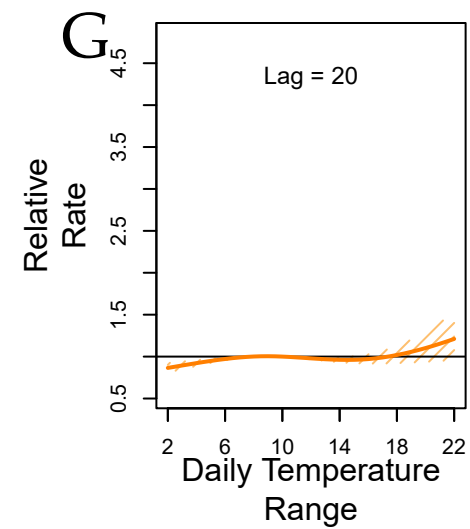

Supplement: S4 Fig — Panel A: The relationship between daily temperature range (x-axis), lag up to 30 days (y-axis), and relative rate of number of mosquitoes caught (z-axis) is plotted. Panel B, D, and F plot slices of the surface along the temperature axis with corresponding uncertainty. Panel C, E, and G plot slices of the surface along the lag axis with corresponding uncertainty. (PDF) [file pntd.0007255.s006.pdf]

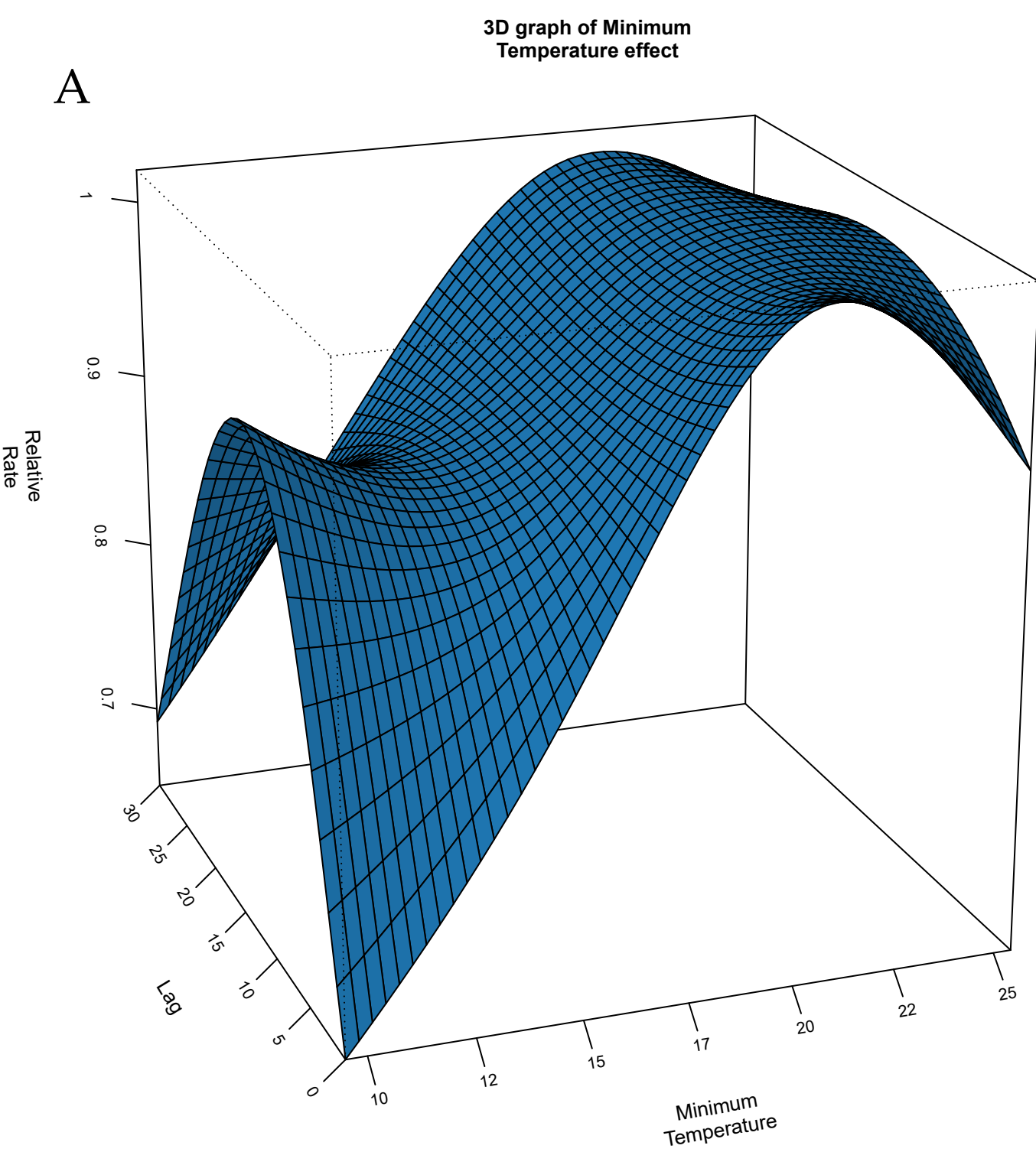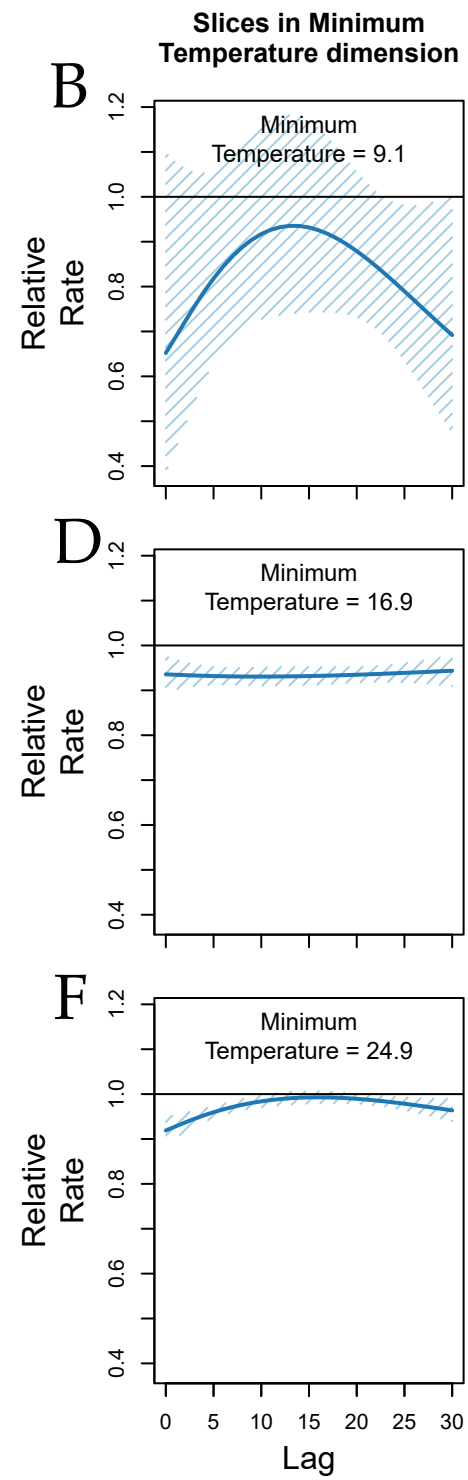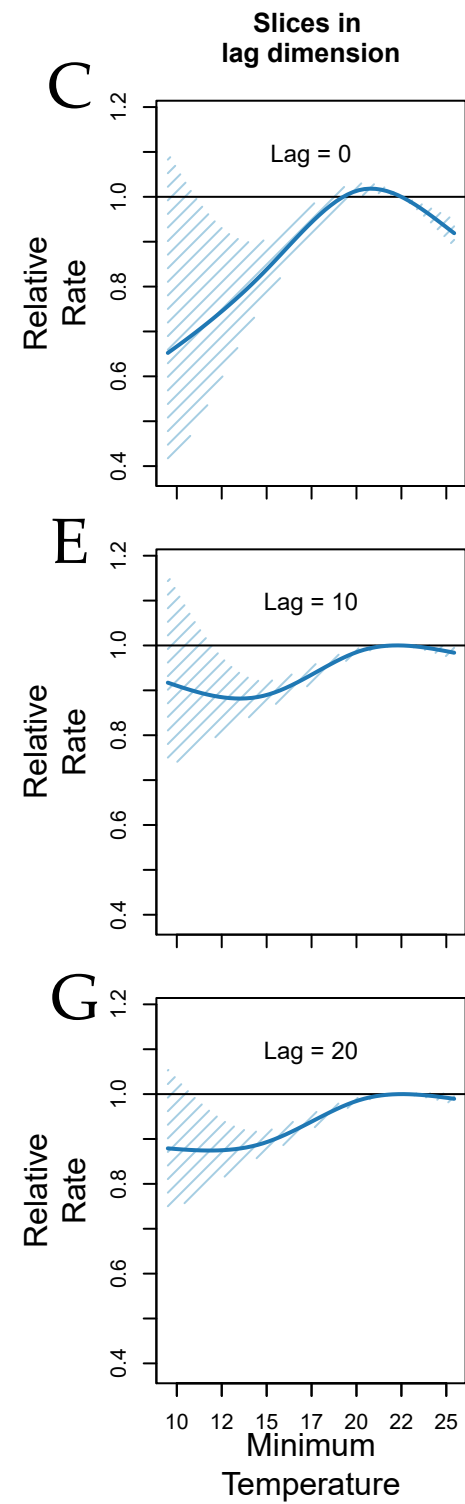

Supplement: S6 Fig — Panel A: The relationship between minimum temperature (x-axis), lag up to 30 days (y-axis), and relative rate of number of mosquitoes caught (z-axis) is plotted. Panel B, D, and F plot slices of the surface along the temperature axis with corresponding uncertainty. Panel C, E, and G plot slices of the surface along the lag axis with corresponding uncertainty. (PDF) [file pntd.0007255.s008.pdf]

3D graph of Precipitation effect

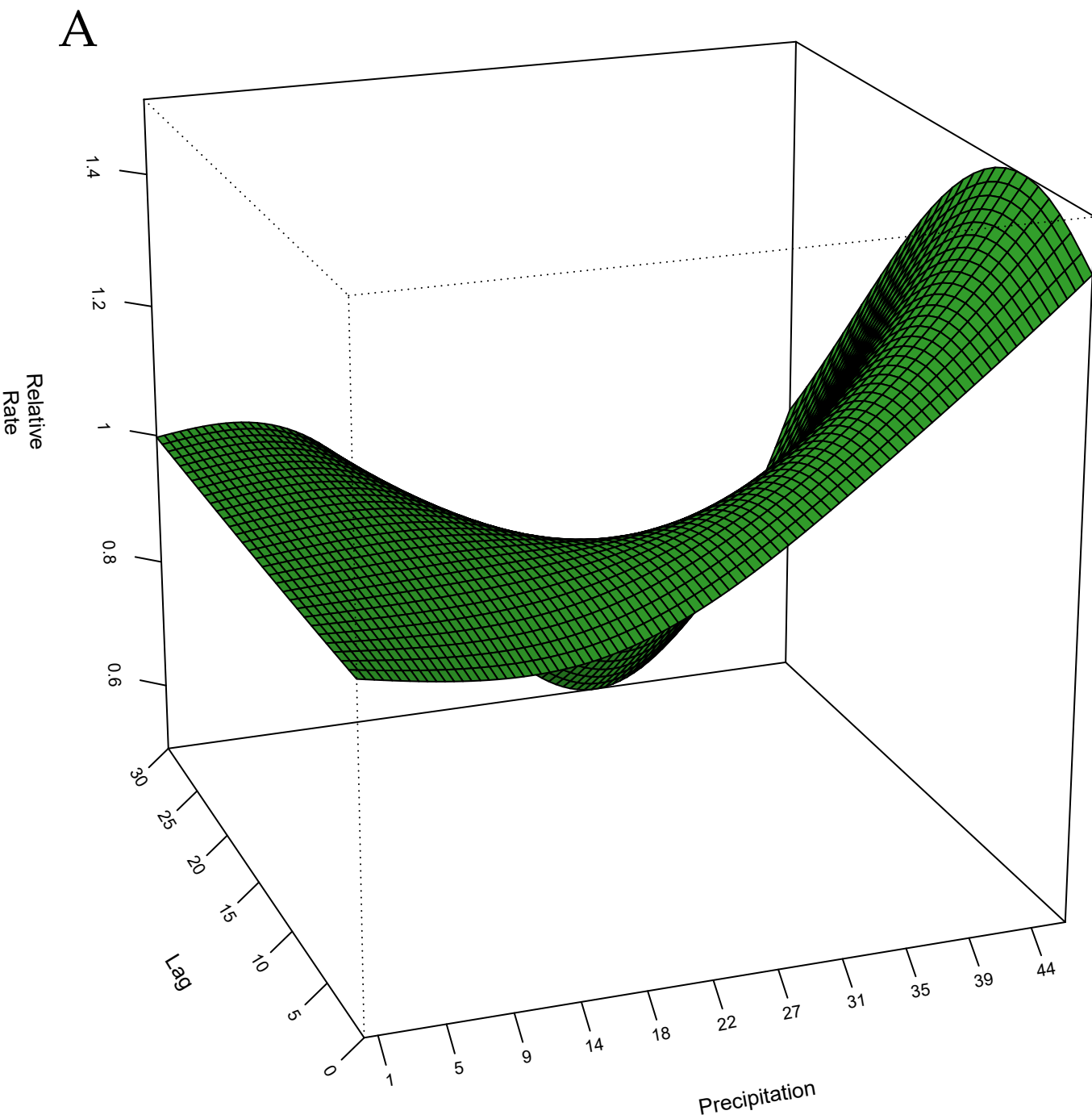

Slices in Precipitation dimension

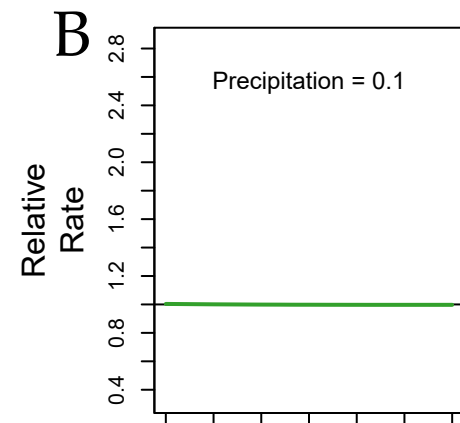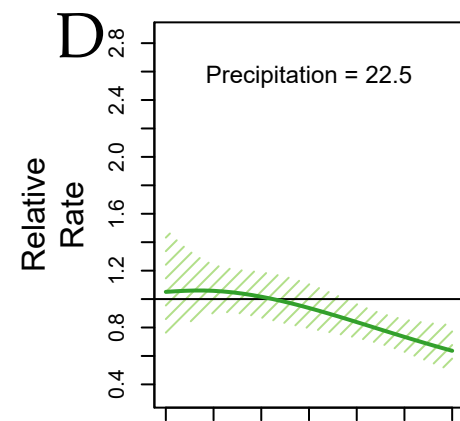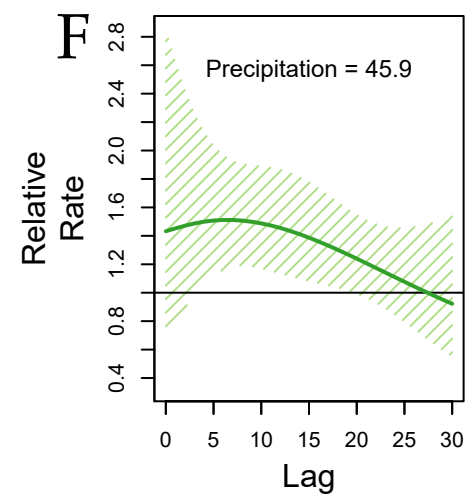

Slices in lag dimension

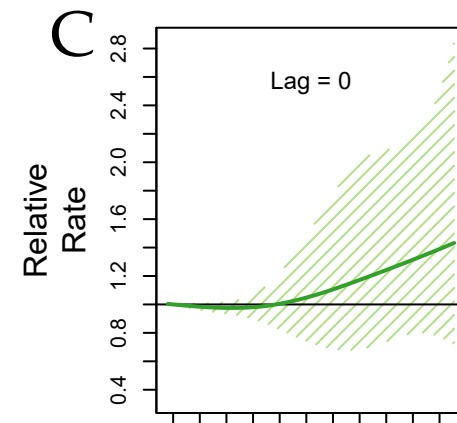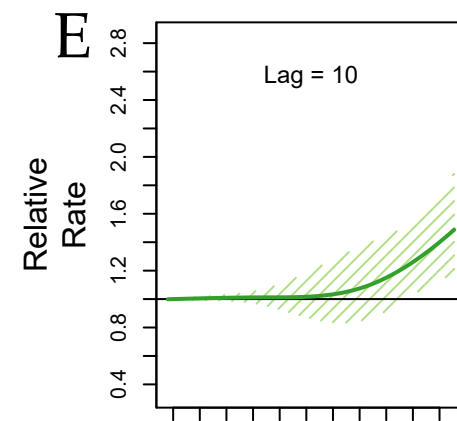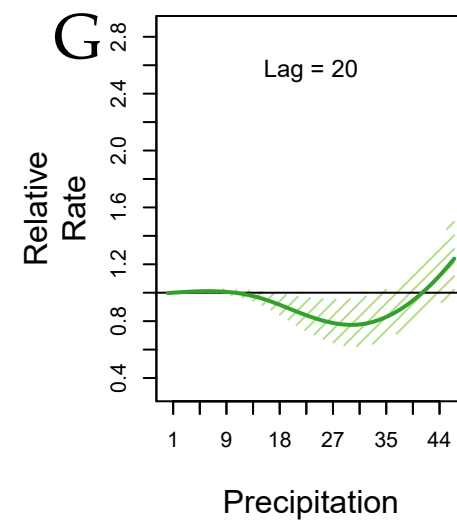

Supplement: S7 Fig — Panel A: The relationship between precipitation (x-axis), lag up to 30 days (y-axis), and relative rate of number of mosquitoes caught (z-axis) is plotted. Panel B, D, and F plot slices of the surface along the temperature axis with corresponding uncertainty. Panel C, E, and G plot slices of the surface along the lag axis with corresponding uncertainty. (PDF) [file pntd.0007255.s009.pdf]

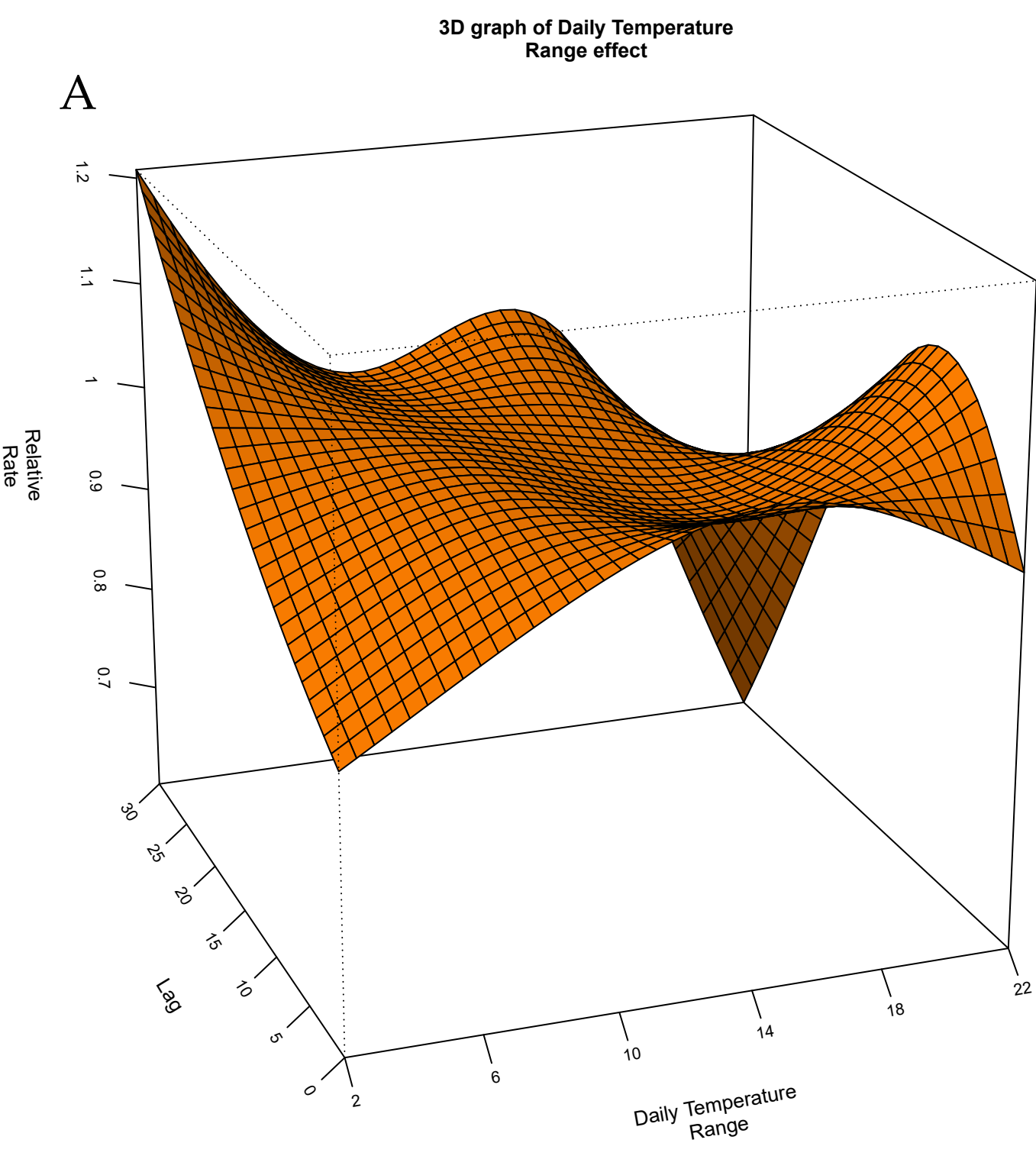

**B**

Slices in Daily Temperature Range dimension

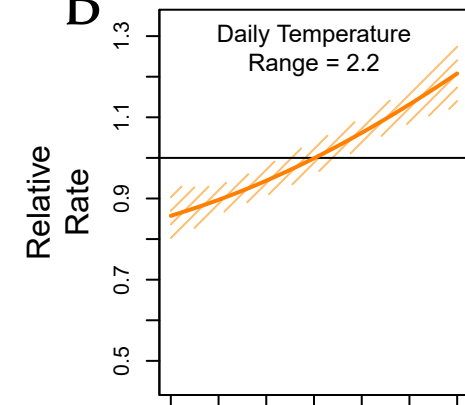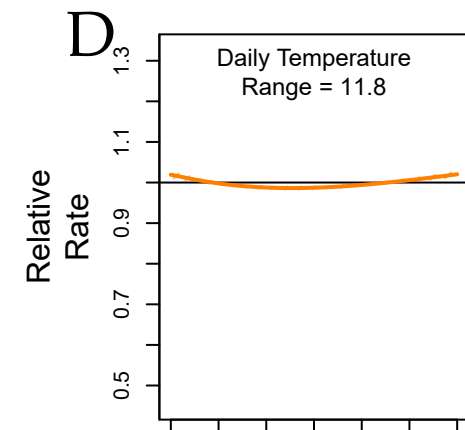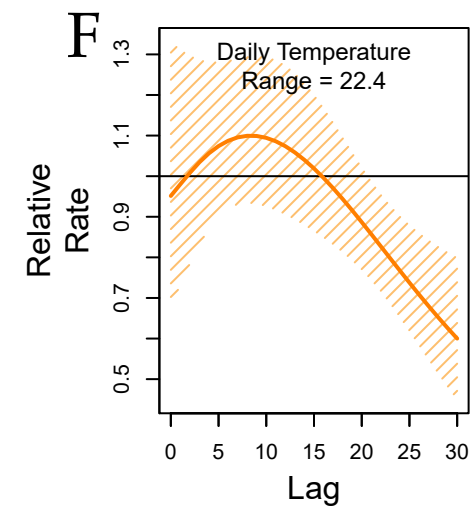

**C**

Slices in lag dimension

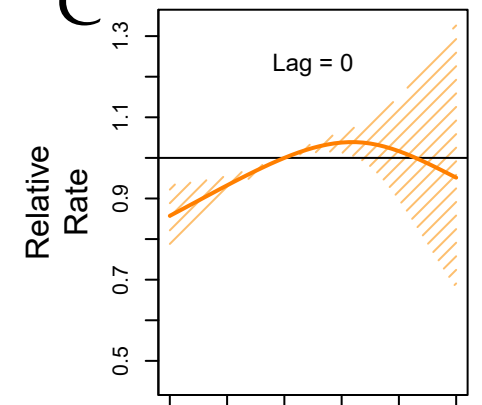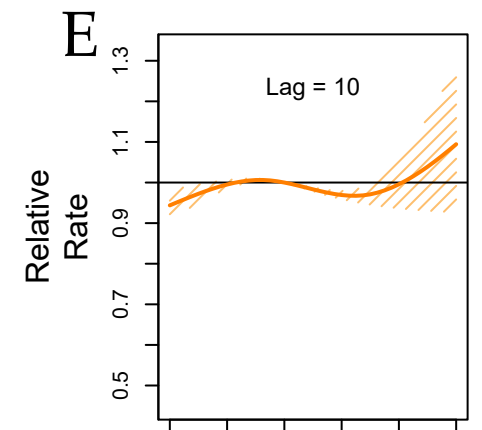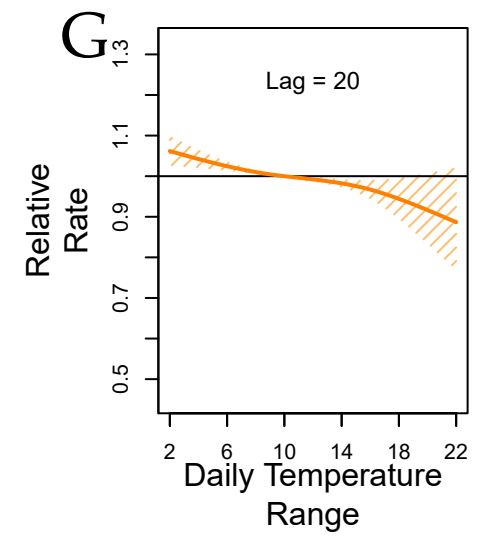

Supplement: S8 Fig — Panel A: The relationship between daily temperature range (x-axis), lag up to 30 days (y-axis), and relative rate of number of mosquitoes caught (z-axis) is plotted. Panel B, D, and F plot slices of the surface along the temperature axis with corresponding uncertainty. Panel C, E, and G plot slices of the surface along the lag axis with corresponding uncertainty. (PDF) [file pntd.0007255.s010.pdf]

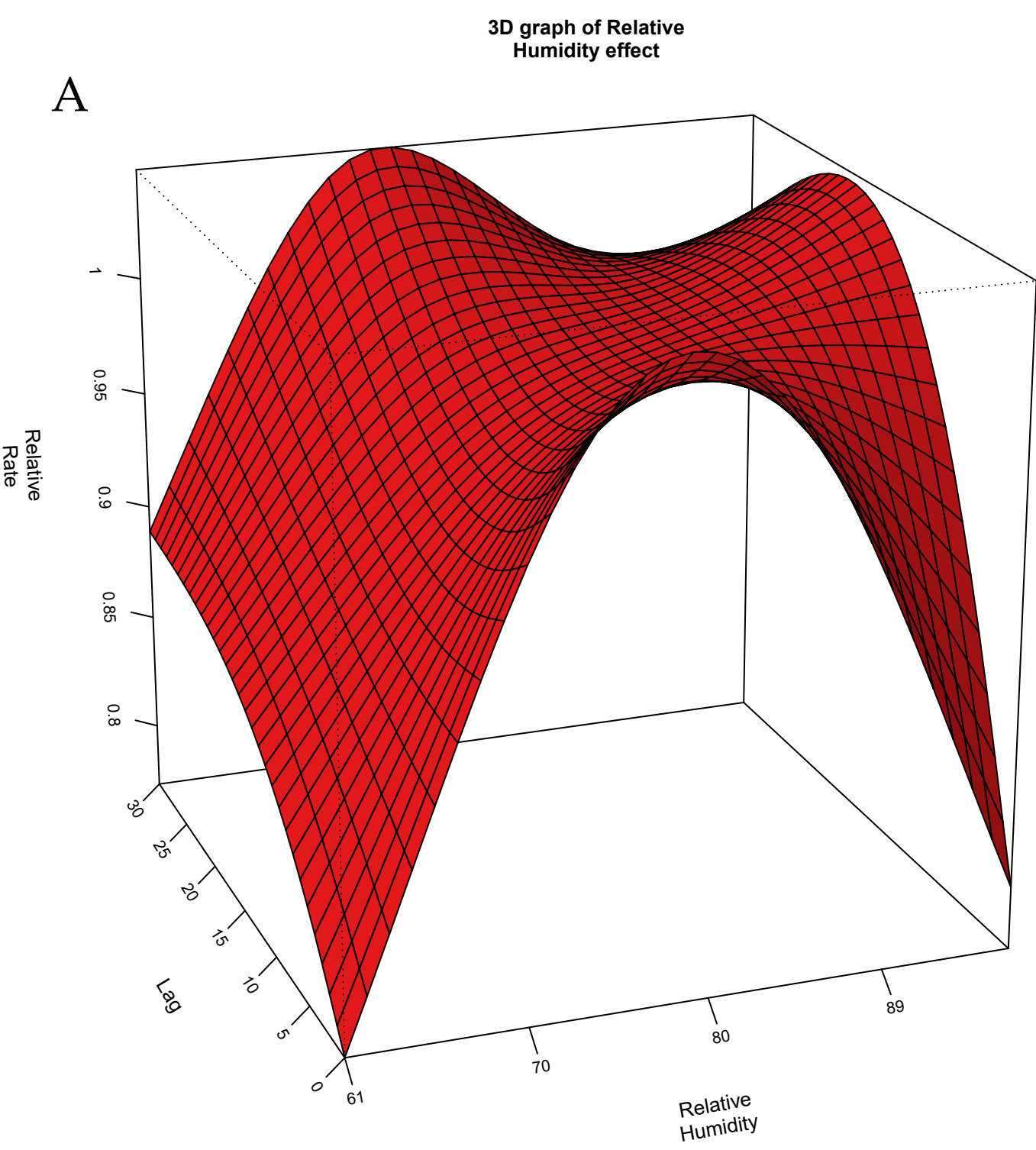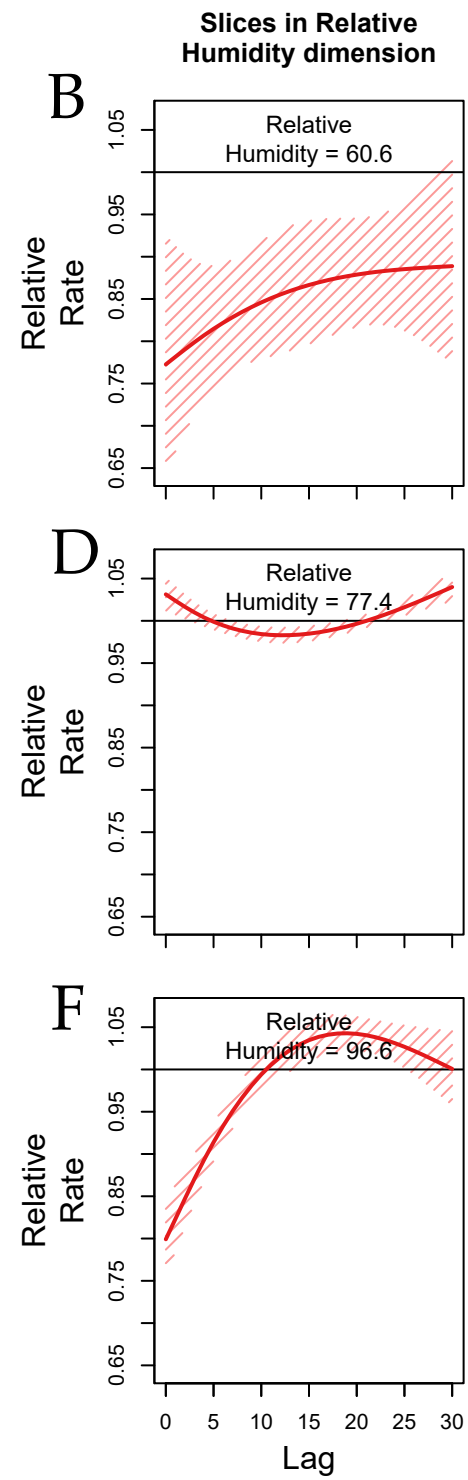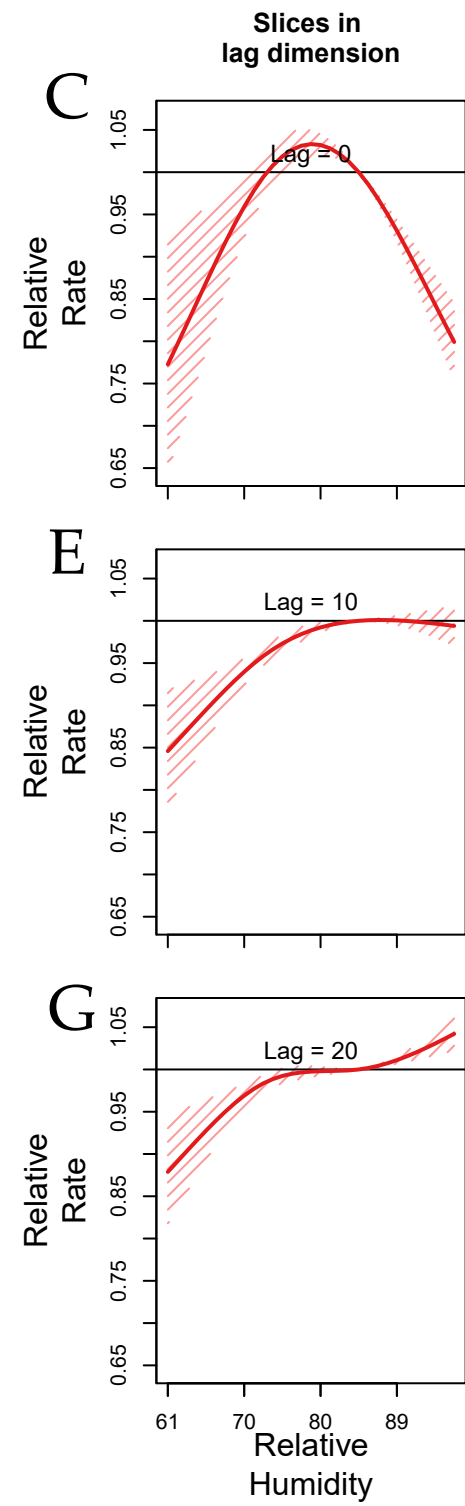

Supplement: S9 Fig — Panel A: The relationship between relative humidity (x-axis), lag up to 30 days (y-axis), and relative rate of number of mosquitoes caught (z-axis) is plotted. Panel B, D, and F plot slices of the surface along the temperature axis with corresponding uncertainty. Panel C, E, and G plot slices of the surface along the lag axis with corresponding uncertainty. (PDF) [file pntd.0007255.s011.pdf]

Mosquito abundance for every home in zone Y

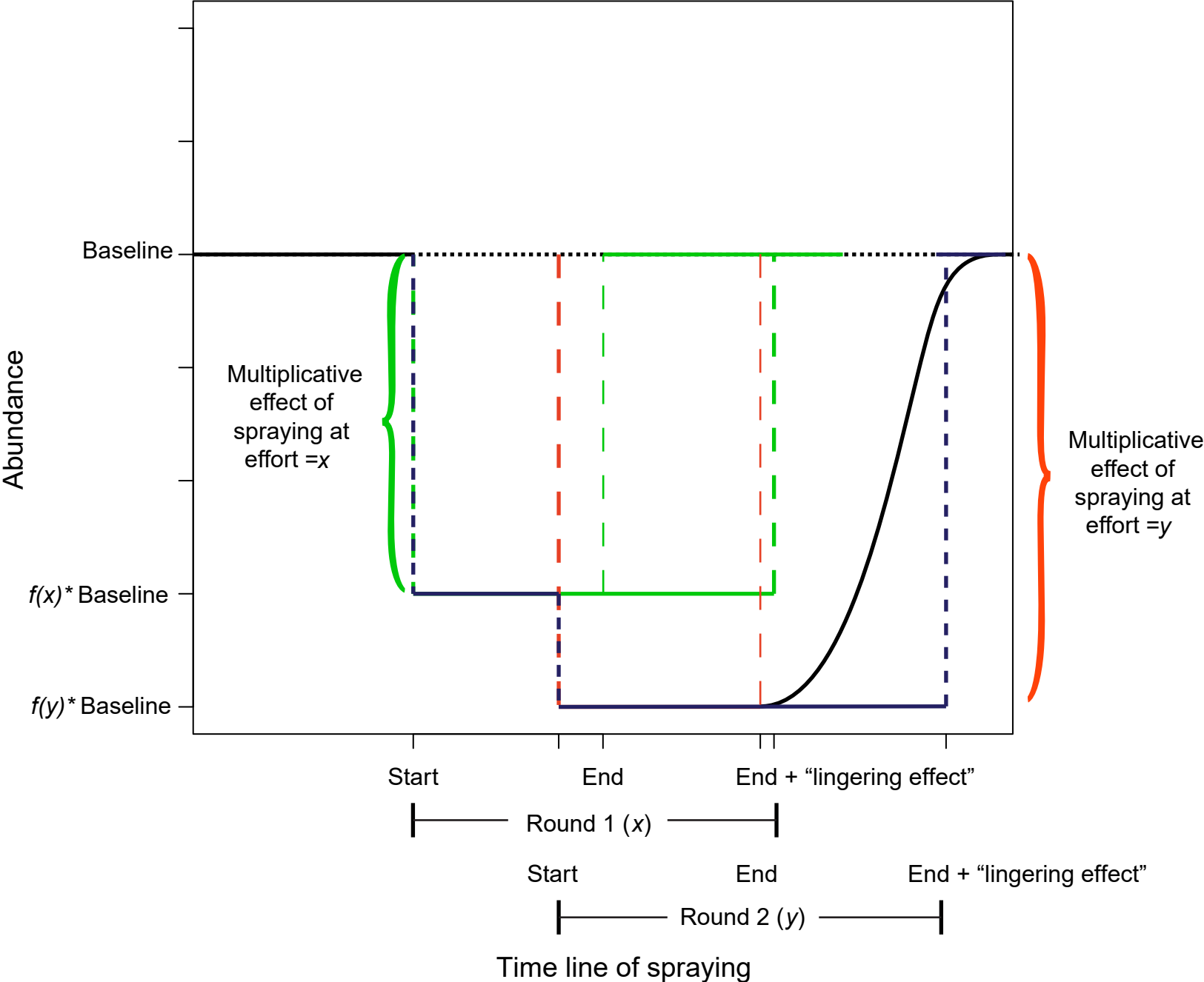

Supplement: S10 Fig — The multiplicative effect of the intervention effort reduces mosquitoes from baseline from the beginning of the intervention round through 3 weeks past the end of the intervention round. If two intervention rounds overlap, the effect is assumed to be the larger of each individual impacts and not additive. The SCAM component of the model fits the function f(x). (PDF) [file pntd.0007255.s012.pdf]

**A** Estimated female AA found  
inside per visit 2001-04-28

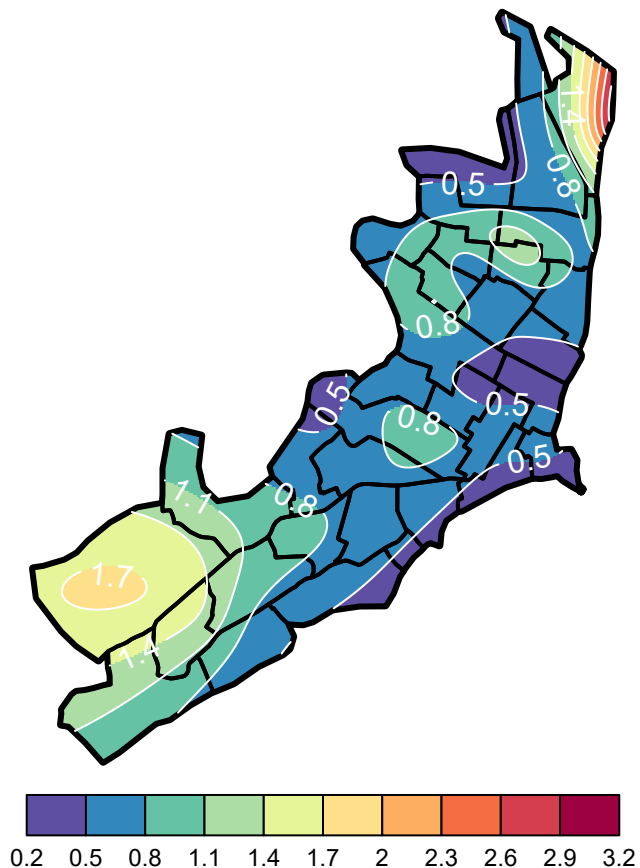

**B** Estimated coefficient of variation  
for 2001-04-28

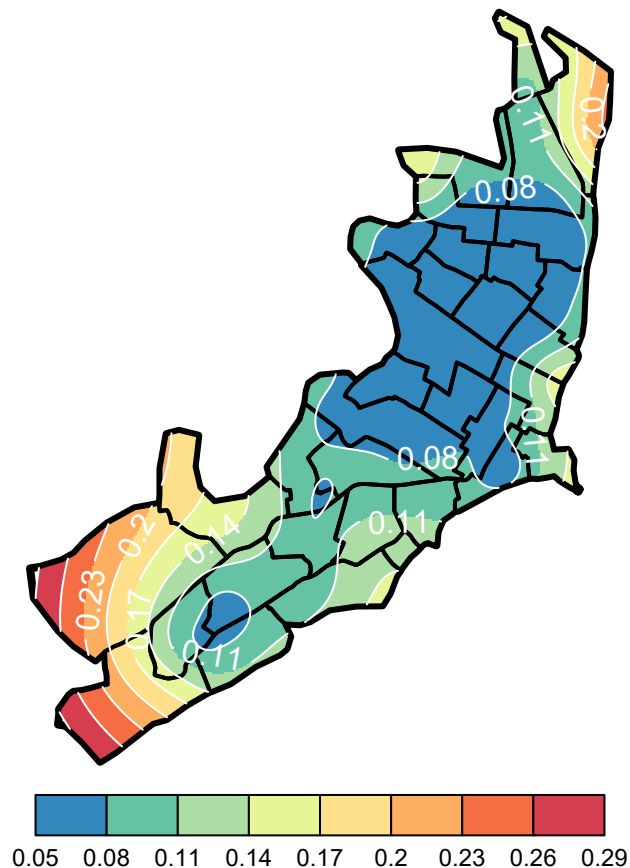

Supplement: S11 Fig — Panel A: Estimated number of female Ae. aegypti that would be caught during a house-hold aspiration held on April 28th, 2001 displayed as a contour plot. The choice of this date was arbitrary but necessary to incorporate the meteorological covariates. Panel B: Estimated coefficient of variation on number of mosquitoes for April 28th, 2001 displayed as a contour plot. (PDF) [file pntd.0007255.s013.pdf]

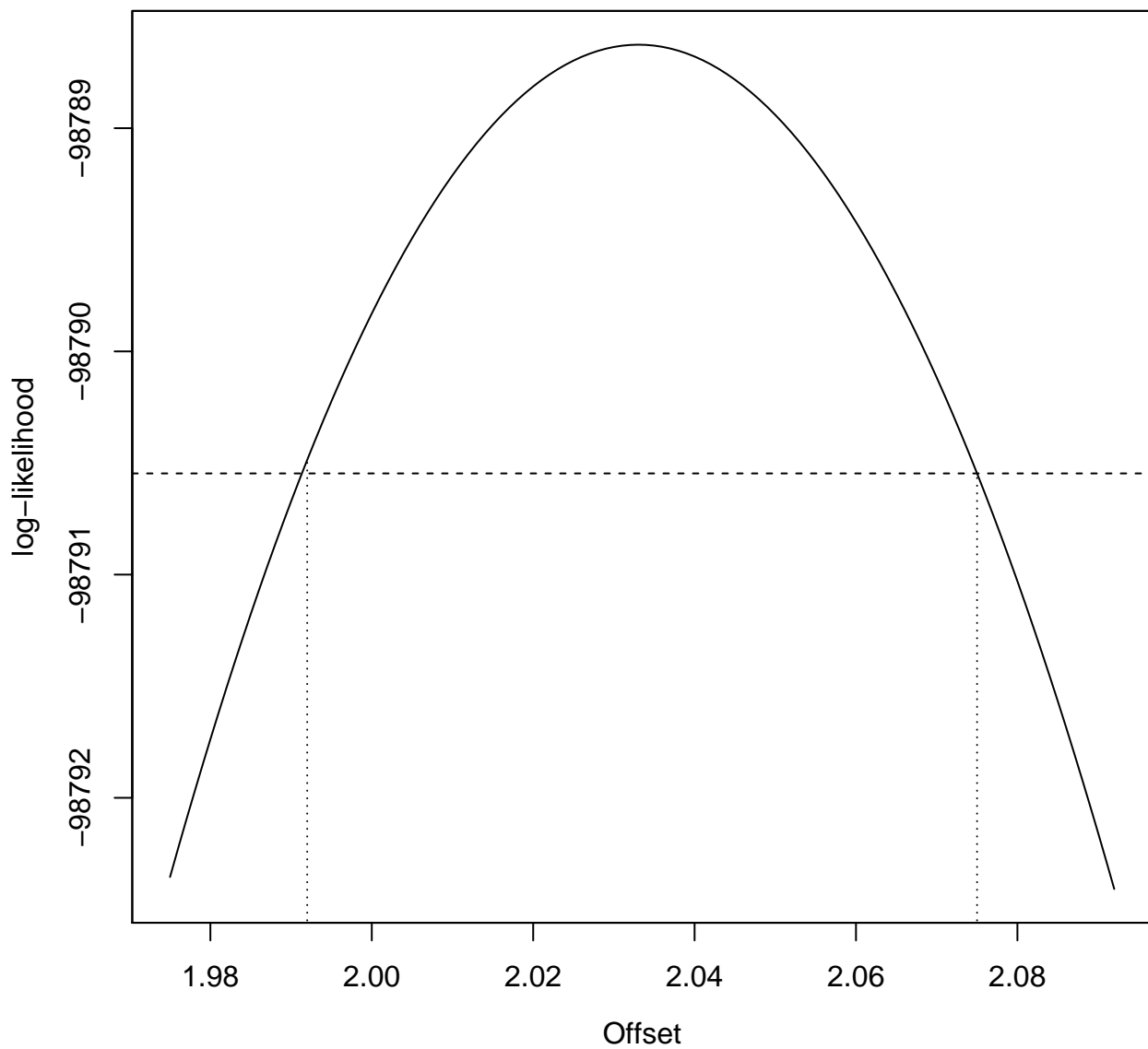

Supplement: S12 Fig — Using the base model, the offset was varied from its maximum likelihood estimate to identify the profile likelihood confidence interval. (PDF) [file pntd.0007255.s014.pdf]

Standard error of log effect of space spray

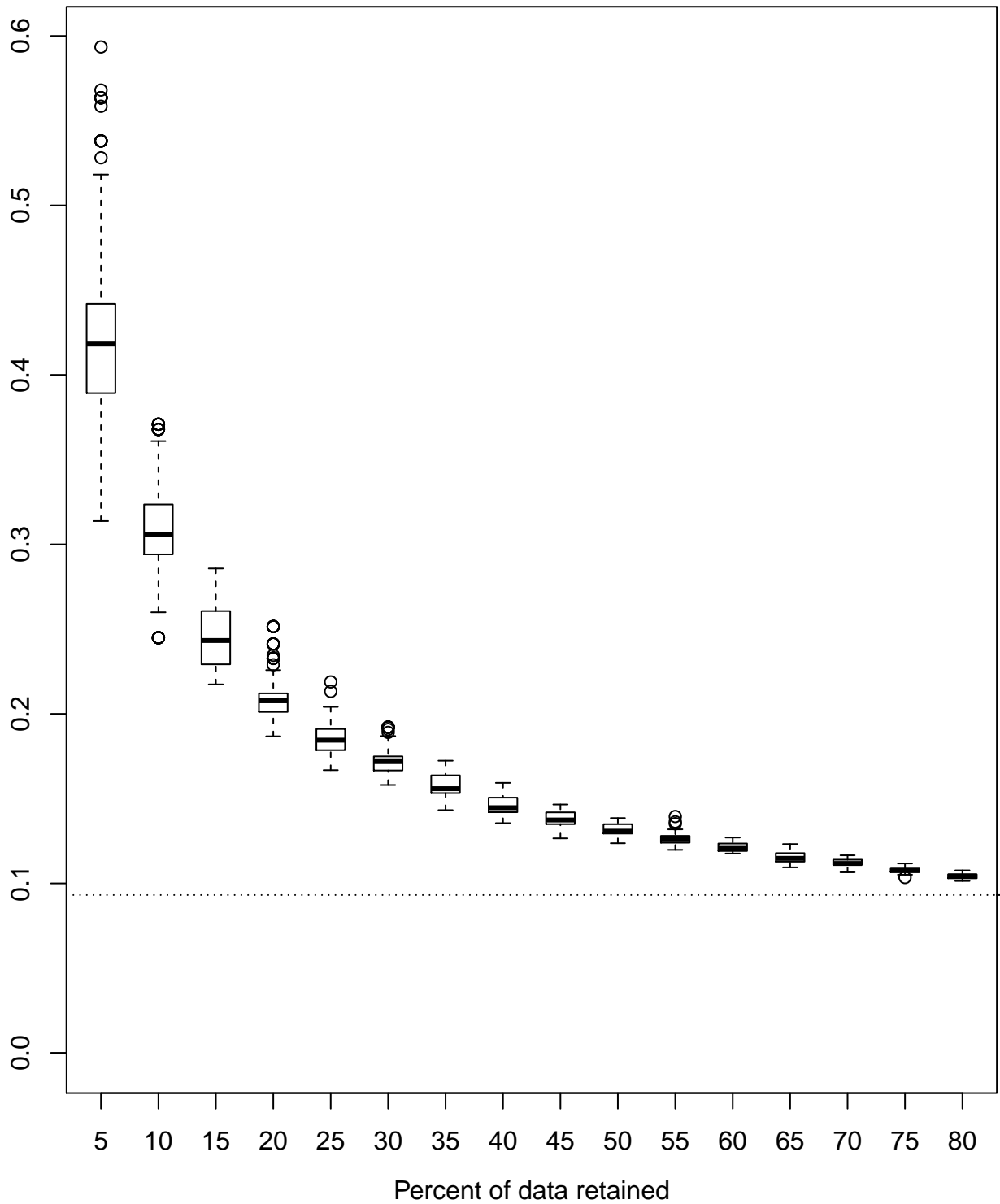

0.09313

Supplement: S14 Fig — The estimated standard error of the effect of space spraying is plotted across 100 experiments for each level of holdout (retaining between 5% and 80% of the original data). The dashed line indicates the fitted standard error of the final model. (PDF) [file pntd.0007255.s016.pdf]

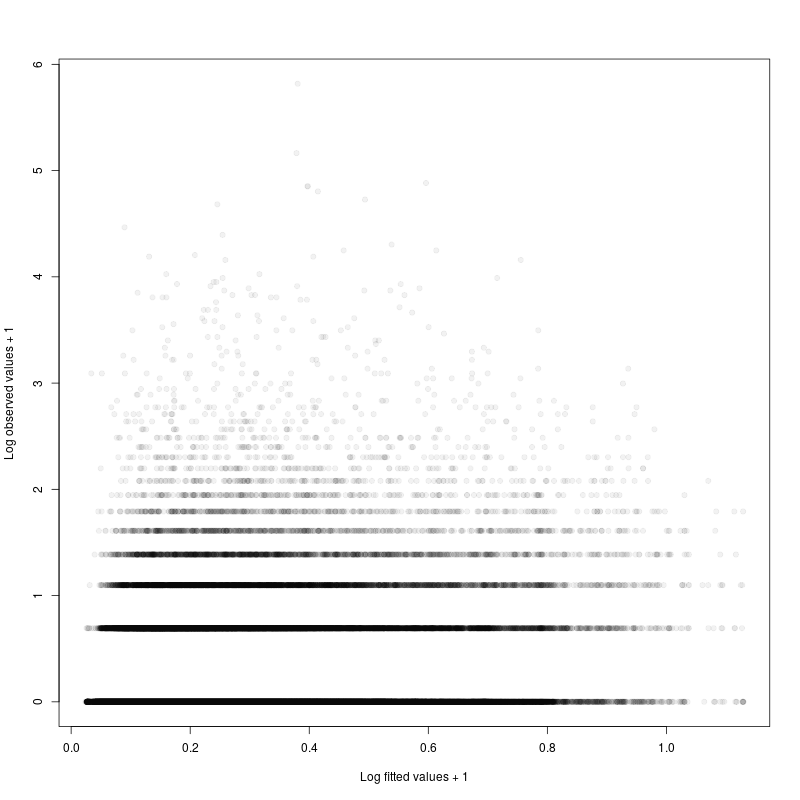

Supplement: S15 Fig — Observations and fitted values are logged (after adding one to each observation) and plotted against each other. (PNG) [file pntd.0007255.s017.png]

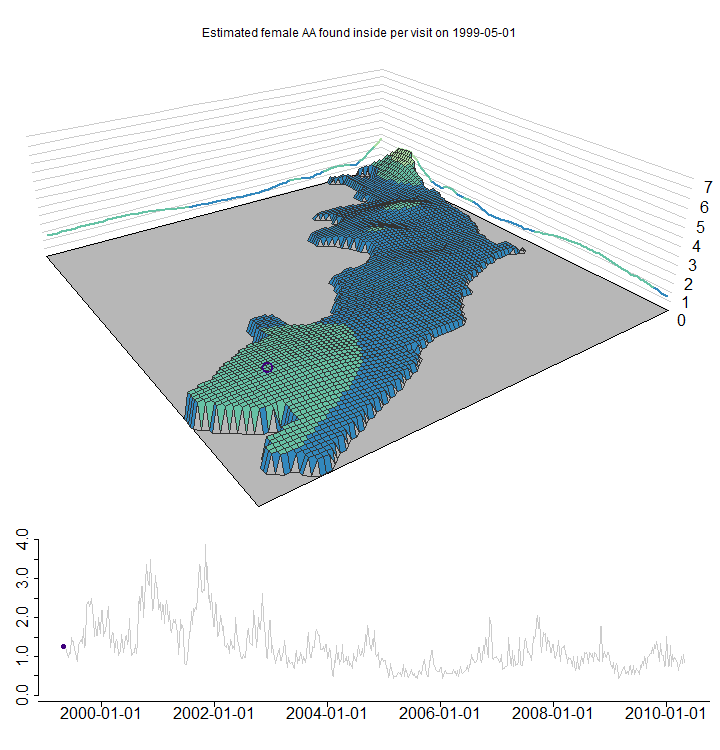

Supplement: S1 Movie — Fig 5A is replotted for the first day of each week across the study period. The time-series is representative of the single point in the South-West of Iquitos indicated by an open circle on the 3-D surface. (GIF) [file pntd.0007255.s018.gif]
