# Supplementary material for: Estimating the impact of city-wide Aedes aegypti population control: An observational study in Iquitos, Peru
Source: PLoS Negl Trop Dis. 2019 May 30;13(5):e0007255. doi: 10.1371/journal.pntd.0007255 (PMC6542505; doi:10.1371/journal.pntd.0007255)
Supplement: S2 Fig — Panel A: The relationship between minimum temperature (x-axis), lag up to 30 days (y-axis), and relative rate of number of mosquitoes caught (z-axis) is plotted. Panel B, D, and F plot slices of the surface along the temperature axis with corresponding uncertainty. Panel C, E, and G plot slices of the surface along the lag axis with corresponding uncertainty. (PDF) [file pntd.0007255.s004.pdf]

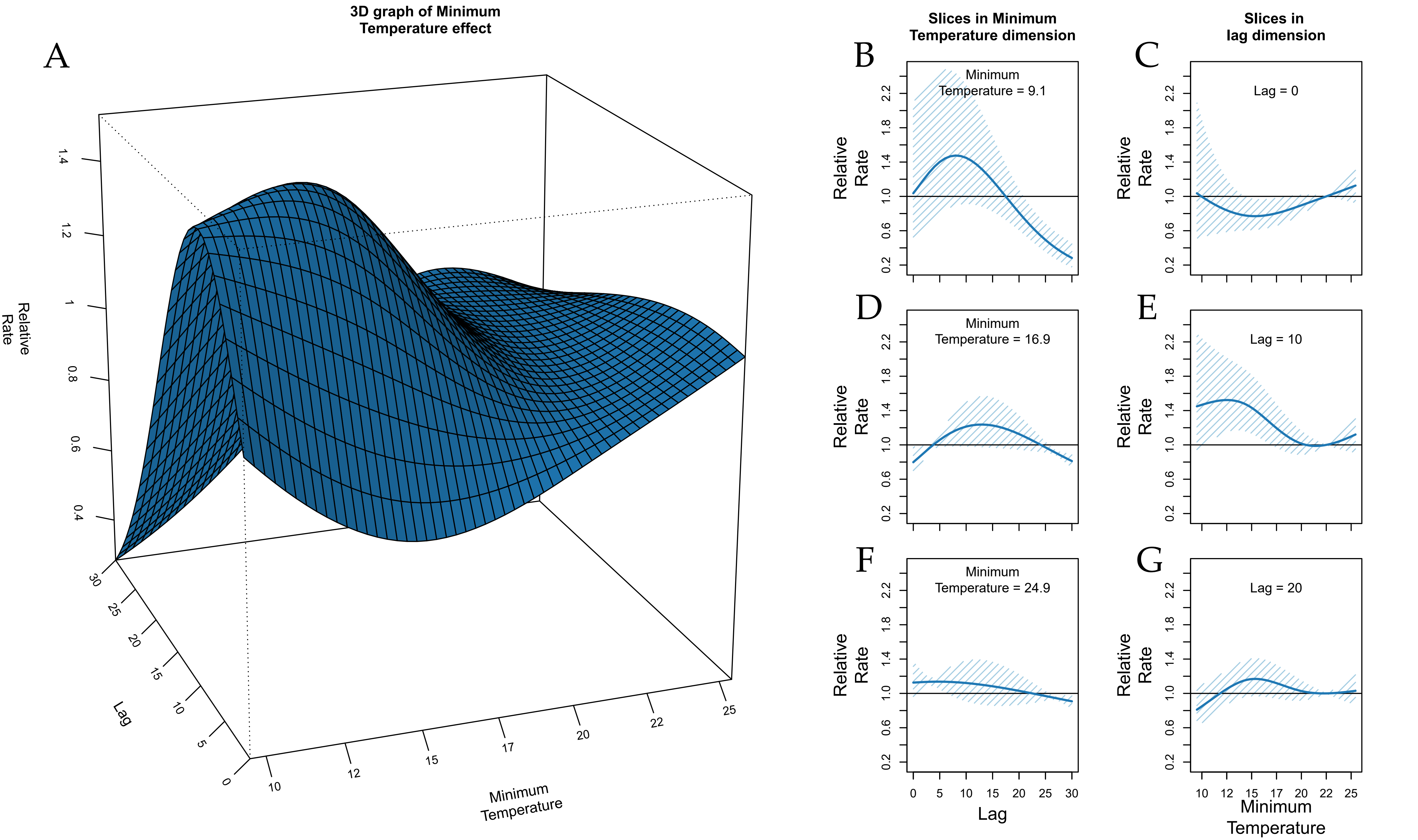

3D graph of Minimum Temperature effect
A
Relative Rate
1.4
1.2
1
0.8
0.6
0.4
Lag
30
25
20
15
10
5
0
Minimum Temperature
10
12
15
17
20
22
25
Slices in Minimum Temperature dimension
Slices in lag dimension
B
Minimum Temperature = 9.1
C
Lag = 0
D
Minimum Temperature = 16.9
E
Lag = 10
F
Minimum Temperature = 24.9
G
Lag = 20
Relative Rate
0.2
0.6
1.0
1.4
1.8
2.2
0
5
10
15
20
25
30
Lag
10
12
15
17
20
22
25
Minimum Temperature
