# Supplementary material for: Estimating the impact of city-wide Aedes aegypti population control: An observational study in Iquitos, Peru
Source: PLoS Negl Trop Dis. 2019 May 30;13(5):e0007255. doi: 10.1371/journal.pntd.0007255 (PMC6542505; doi:10.1371/journal.pntd.0007255)
Supplement: S5 Fig — Panel A: The relationship between relative humidity (x-axis), lag up to 30 days (y-axis), and relative rate of number of mosquitoes caught (z-axis) is plotted. Panel B, D, and F plot slices of the surface along the temperature axis with corresponding uncertainty. Panel C, E, and G plot slices of the surface along the lag axis with corresponding uncertainty. (PDF) [file pntd.0007255.s007.pdf]

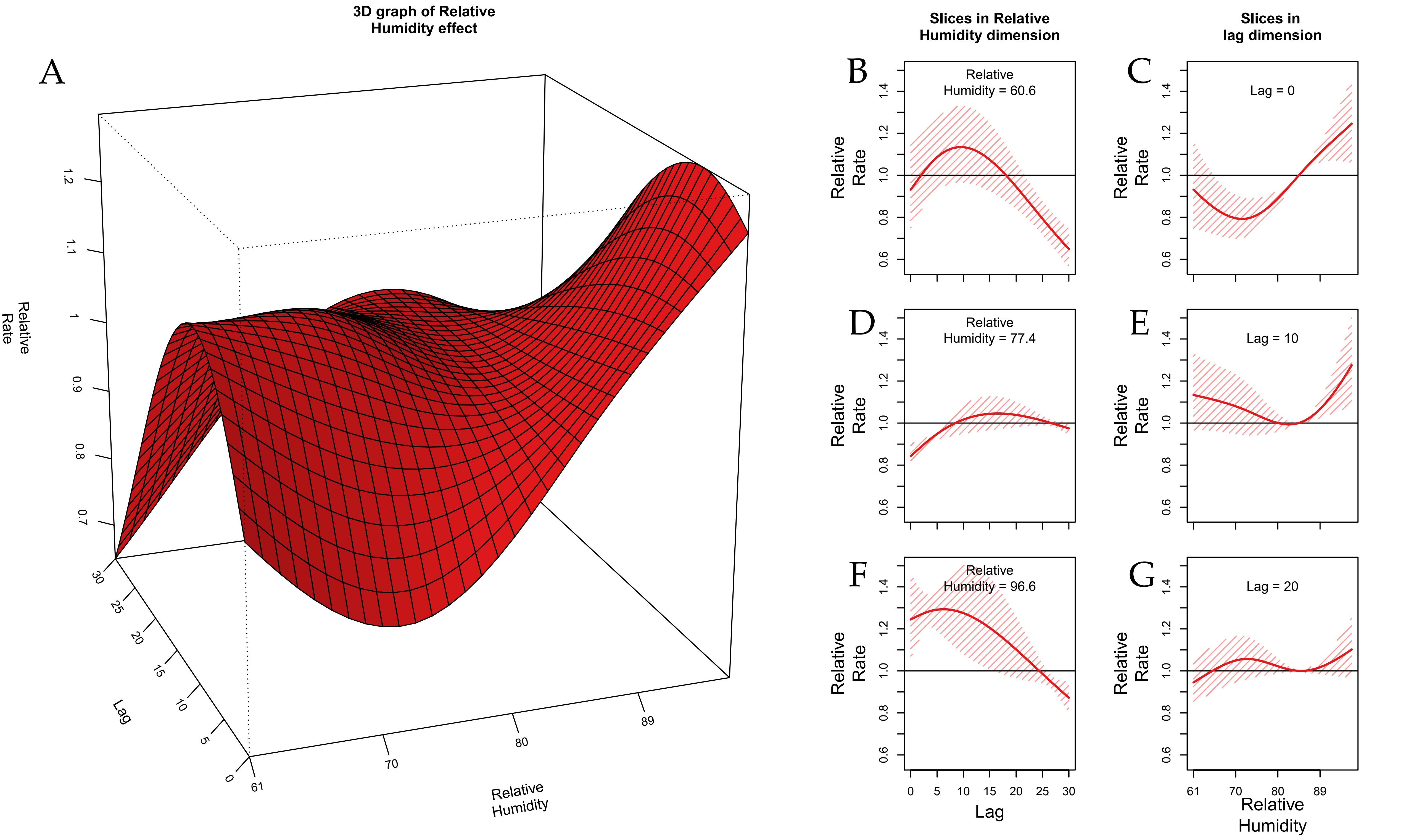

3D graph of Relative Humidity effect
A
Relative Rate
1.2
1.1
1
0.9
0.8
0.7
Lag
30
25
20
15
10
5
0
61
70
80
89
Relative Humidity
Slices in Relative Humidity dimension
Slices in lag dimension
B
Relative Humidity = 60.6
C
Lag = 0
D
Relative Humidity = 77.4
E
Lag = 10
F
Relative Humidity = 96.6
G
Lag = 20
Relative Rate
0.6
0.8
1.0
1.2
1.4
0 5 10 15 20 25 30
Lag
61 70 80 89
Relative Humidity
