# Supplementary material for: Estimating the impact of city-wide Aedes aegypti population control: An observational study in Iquitos, Peru
Source: PLoS Negl Trop Dis. 2019 May 30;13(5):e0007255. doi: 10.1371/journal.pntd.0007255 (PMC6542505; doi:10.1371/journal.pntd.0007255)
Supplement: S13 Fig — The estimated effect of space spraying is plotted across 100 experiments for each level of holdout (retaining between 5% and 80% of the original data). The left y-axis shows the fitted value in log space, while the right y-axis displays it in natural space. The dashed line indicates the fitted value of the final model. (PDF) [file pntd.0007255.s015.pdf]

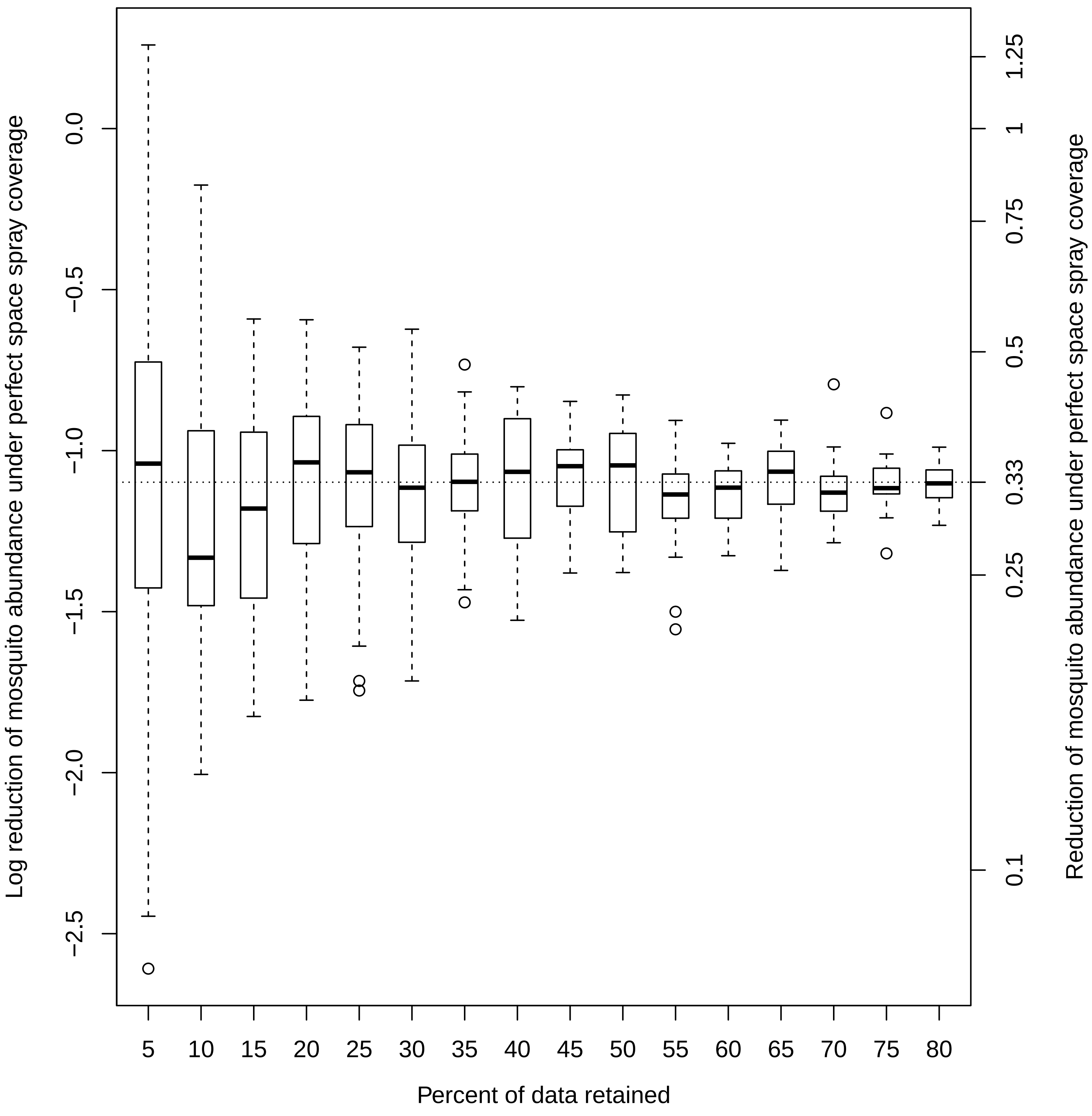

Log reduction of mosquito abundance under perfect space spray coverage
0.0
−0.5
−1.0
−1.5
−2.0
−2.5
Reduction of mosquito abundance under perfect space spray coverage
1.25
1
0.75
0.5
0.33
0.25
0.1
5 10 15 20 25 30 35 40 45 50 55 60 65 70 75 80
Percent of data retained
